# Impaired JAK-STAT pathway signaling in leukocytes of the frail elderly

**DOI:** 10.1186/s12979-021-00261-w

**Published:** 2022-01-17

**Authors:** Leonard Daniël Samson, Peter Engelfriet, W. M. Monique Verschuren, H. Susan J. Picavet, José A. Ferreira, Mary-lène de Zeeuw-Brouwer, Anne-Marie Buisman, A. Mieke H. Boots

**Affiliations:** 1 National Institute of Public Health and the Environment, Bilthoven, The Netherlands; 2 Department of Rheumatology and Clinical Immunology, University of Groningen, University Medical Center Groningen, Groningen, The Netherlands; 3 Julius Center for Health Sciences and Primary Care, University Medical Center Utrecht, Utrecht University, Utrecht, The Netherlands

**Keywords:** Immunosenescence, Frailty, JAK-STAT pathway, Immune function, Phospho-flow cytometry, Chronic low-grade inflammation

## Abstract

**Background:**

Elderly often show reduced immune functioning and can develop chronic low-grade inflammation. Why some elderly are more prone to become frail is unknown. We investigated whether frailty is associated with altered cytokine signaling through the JAK-STAT pathway in leukocytes of 34 individuals aged 65–74 years. In addition, we investigated how this relation is affected by chronic low-grade inflammation during the previous 20 years. Cytokine signaling was quantified by measuring intracellular STAT1, STAT3, and STAT5 phosphorylation in monocytes, B cells, CD4^+^ T cells and CD8^+^ T cells upon stimulation with IL-2, IL-6, IL-10, IFNα and IFNγ, using phospho-flow cytometry. Presence of chronic low-grade inflammation was investigated by evaluating 18 different plasma inflammatory markers that had been measured repeatedly in the same individuals over the previous 20 years. Frailty was assessed as a score on a frailty index.

**Results:**

We found that lower cytokine-induced pSTAT responsiveness in the various cell subsets was seen with higher frailty scores in both men and women, indicative of dysfunctional pSTAT responses in frailer individuals. Associations differed between men and women, with frailer women showing lower pSTAT1 responses in monocytes and frailer men showing lower pSTAT5 responses in CD4^+^ and CD8^+^ T cells. Notably, lower IL-10-induced pSTAT3 responses in men were related to both higher frailty scores and higher CRP levels over the past 20 years. This might indicate poor resolution of low-grade inflammation due to defective regulatory pSTAT signaling in older men.

**Conclusions:**

Our results emphasize the importance of preserved JAK-STAT pathway signaling in healthy aging and reveal cellular pSTAT levels as a candidate biomarker of frailty.

**Supplementary Information:**

The online version contains supplementary material available at 10.1186/s12979-021-00261-w.

## Introduction

Adequate functioning of the immune system is thought to be a pivotal factor in the healthy aging process [1]. Older people tend to have less adequate immune responses to infections, including in particular respiratory infections such as COVID-19 [2]. Also vaccine responses generally are weaker [3, 4], as are immune functions for surveillance and clearing of (pre)malignant cells [5]. An important sign of immune dysregulation is the presence of a ‘sterile’ low-grade chronic inflammation, more often seen in older individuals [6–8]. It is thought that this low-grade inflammation coincides with a reduced functioning of immune cells, especially that of macrophages, leading to a poor clearance of accumulating tissue debris and senescent cells from the body [9]. Also, other major innate and adaptive immune cell subsets, such as CD4^+^ and CD8^+^ T cells, and B cells, are essential for proper immune signaling and could thus be involved in reduced functioning of the immune system. Furthermore, senescent cells could directly contribute to chronic low-grade inflammation since they produce multiple inflammatory cytokines (known as the Senescence Associated Secretory Profile, SASP), and these cells are more abundant in older people [10].

An important factor relevant to reduced immune responses in older people could be impaired cellular cytokine signaling. Many cytokines are known to signal through the Janus kinase-Signal Transducer and Activator of Transcription proteins (JAK-STAT) pathway. In this pathway, cytokines (mainly interleukins and interferons) bind to surface receptors causing a chain reaction which ultimately leads to the intracellular phosphorylation of STAT proteins. This, in turn, can induce transcription of genes leading to adequate immune responses [11]. Loss-of-function mutations within the JAK-STAT pathway are related to life-threatening diseases such as severe combined immunodeficiency [12], and to increased susceptibility to and severity of infections [13, 14]. Conversely, overactivation of the JAK-STAT pathway is also linked to dysfunctional immune responses. This is currently of particular interest since recently developed JAK inhibitors were shown to be effective in the treatment of auto-immune diseases such as ulcerative colitis [15] and rheumatoid arthritis [16].

JAK-STAT signaling was found to be impaired in older people, with reduced signaling [17], and higher baseline cellular STAT activation [17, 18]. Also, results of preclinical studies suggest that low-grade inflammation in older people can be reduced with JAK inhibitors [19, 20]. The immune system in elderly may also be dysregulated due to inadequate responses to triggering of pattern-recognition receptors, such as toll-like receptors (TLR). These receptors can recognize pathogens or parts of pathogens and activate immune cells, which can in turn produce pro-inflammatory cytokines. Diminished TLR responses have been reported with higher age in most studies [21, 22] although not in a study that investigated strictly healthy older participants [23].

To better understand the role of cellular signaling in the aging process, it is important to know if reduced cellular immune responses are seen in all elderly or only in those that are frail. Frailty can be assessed by means of a frailty index score; people with a high score have been shown to have an increased risk of ‘adverse’ life events (e.g. injury) and a reduced capacity to recover from these events [24, 25].

In this study we investigated whether diminished immune cellular responses in older men and women are related to frailty and if impaired cytokine signaling is related to more pronounced chronic low-grade inflammation. We analyzed cytokine-induced cellular signaling through the JAK-STAT pathway and studied cellular activation through the TLR pathway in 34 individuals selected from a Dutch longitudinal population-based cohort study. The presence of chronic low-grade inflammation was evaluated by analysis of 18 different inflammatory markers in blood samples that were repeatedly taken from the same individuals at 5 year intervals over the past 20 years, between 1991 and 2017.

## Results

### Study population characteristics

**Fig. 1:**
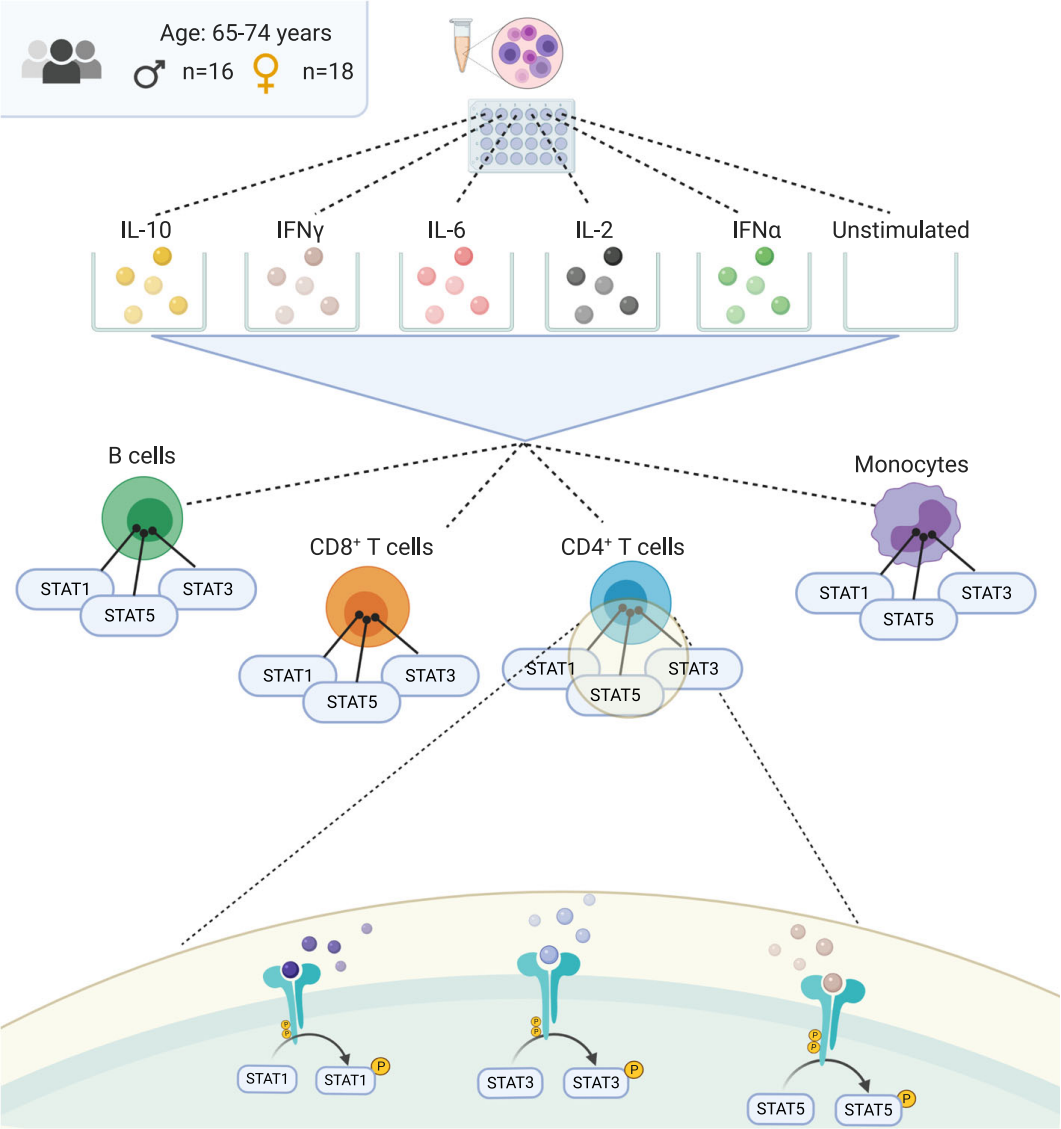
Study and experimental design. CMV-seronegative participants were selected from the Doetinchem cohort study. PBMCs were thawed and stimulated with the indicated cytokines or were left unstimulated (baseline). Intranuclear immunofluorescence staining was performed to quantify phosphorylation of STAT1, 3, and 5 proteins, in combination with surface staining for lineage-specific markers to distinguish between B cells, monocytes, and T cells (CD8^+^ and CD4^+^)

The 34 participants were 65–74 years old (Fig. 1), selected from the Doetinchem cohort study (DCS), a longitudinal study that started in 1987 with the participants being followed up ever since [26, 27]. CMV seropositive individuals were excluded in order to avoid CMV infection being a confounder in the study. The median frailty index score of the participants was 0.09, ranging from 0 (0/36 deficits present) to 0.4 (14/36 deficits) (Table 1). The mean BMI level was 26.9 kg m^− 2^ and ranged from 20.8–35 kg m^− 2^. Frailty index scores (see Table S1 for frailty index components), BMI levels and age did not differ significantly between men and women (Fig. S1A-C).

**Table 1:** Baseline characteristics of the study population (*n* = 34)

% (n) of women	52.9 (18)
Age, yrs.	69.4 (SD: 2.6, range: 65.4–74.1)
BMI, kg m^− 2^	26.9 (SD: 3.4, range: 20.8–35)
Frailty index score	0.09 (0.18, range: 0–0.4)
CMV-, %	100

Frailty index score: health score (median + interquartile range) enumerating 36 health deficits, theoretically ranging from 0 (no deficits) to 1 (all deficits). BMI and age are in mean + standard deviation. CMV-seronegative study participants were selected for this study. % of women: percentage of women of the total study population

### Cytokine-induced pSTAT responsiveness within different immune cell lineages

**Fig. 2:**
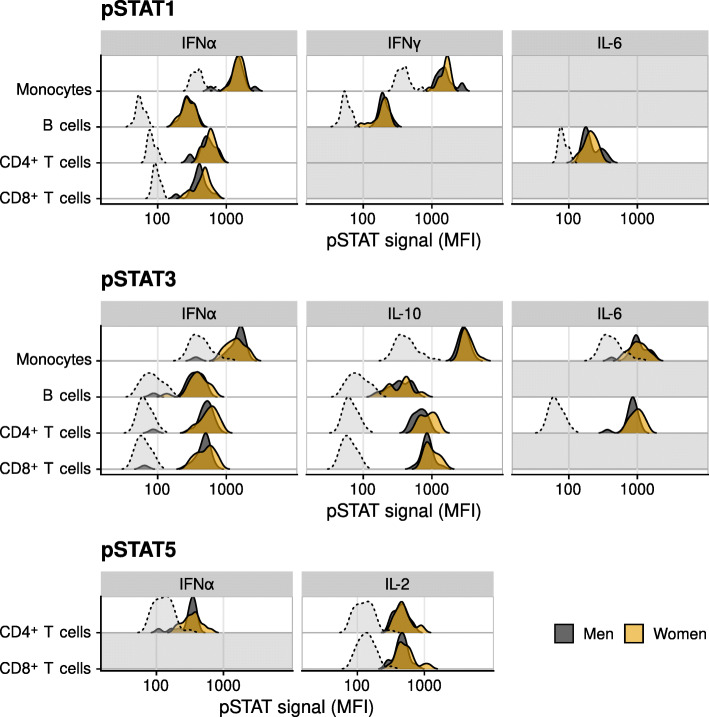
Levels of phosphorylated STAT proteins (pSTAT1, pSTAT3, and pSTAT5), measured in CD4^+^ T cells, CD8^+^ T cells, B cells, and monocytes. Grey surface area with dashed outline shows baseline (unstimulated) pSTAT levels. Dark grey and orange surface areas with solid outline show pSTAT immune cell responses to stimulation in men and women, respectively. Shown are 20 out of 60 experimental stimuli conditions that induced a robust change (fold change > 2) compared to the baseline condition in CD4^+^, CD8^+^, B cells and monocytes

We used a broad panel of experimental conditions to quantify immune cell cytokine responses by separately stimulating PBMCs with IL-10, IFNγ, IL-6, IL-2, or IFNα, and subsequently measuring phosphorylated STAT1, STAT3, and STAT5 (pSTATs) in monocytes, B cells, and CD4^+^ and CD8^+^ T cells (Fig. 1), using phosho-flow cytometry (Fig. S2). Examples of individual pSTAT expression patterns after stimulation can be found in Fig. S3. Baseline (unstimulated) levels of pSTAT1, pSTAT3, and pSTAT5 were higher in monocytes than in the other cell subsets, and those of pSTAT5 were higher than those of pSTAT1 and pSTAT3 in both CD4^+^ and CD8^+^ T cells. (Fig. S4, light grey density plots). The baseline pSTAT levels did not differ significantly between men and women (Table S2). Monocytes showed relatively high induced pSTAT levels (after stimulation), besides high baseline pSTAT levels. As expected, not all experimental conditions induced responses of pSTAT1, pSTAT3 and pSTAT5; therefore we selected the conditions showing a ‘clear’ response upon stimulation (defined as a median fold change ≥2) for further analysis (Fig. 2, Fig. S5, 20 out of 60 stimulation conditions). Most of such responses were induced by IFNα, which is indeed expected to induce not only phosphorylation of STAT1, but also STAT3 and pSTAT5 through the type I IFN receptor [17, 28, 29]. Other responses described in literature that we observed were IL2-STAT5 responses in T cells [12], IFNγ-STAT1 responses in monocytes and B cells [12], and IL6- and IL10- induced STAT3 responses in monocytes and CD4^+^ T cells. IL6 also induced STAT1 in CD4^+^ T cells, which has been shown before [30]. The highest responses were seen in CD4^+^ and CD8^+^ T cells after stimulation with IL-10, with a fold change in pSTAT3 levels of 11.9 and 15.2, respectively (Table S3). When testing for sex-specific differences, we did not observe differences in baseline pSTAT levels between men and women, but we did find that women had higher pSTAT3 responses to IL-10 stimulation in CD4^+^ T cells and to some extent also in CD8^+^ T cells, although the differences were small (Table S4, Fig. 2). Next, we examined how cell subset numbers differed between the sexes and with frailty in this population, since a difference in cell numbers might be a confounder in our analyses. We observed that women on average had higher numbers of B cells but lower numbers of monocytes than men (Fig. S6A). In line with our previous data [31], monocyte numbers in women, but not men, were higher with higher frailty index scores (Fig. S6B).

### Frailty is associated with lower cellular pSTAT responsiveness

**Fig. 3:**
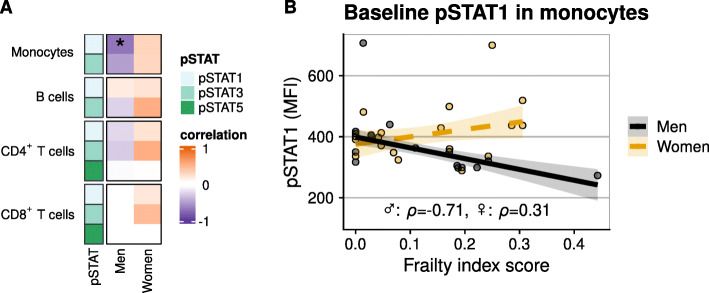
**A** Heatmap showing correlation coefficients of the relation between frailty and the baseline (unstimulated) phosphorylation of STAT1, STAT3, and STAT5 in monocytes, B cells, and CD4^+^ and CD8^+^ T cells. Every box displays the Spearman’s *ρ* value based on data of 16 men and 18 women. **B** Baseline (unstimulated) pSTAT1 expression in monocytes. Solid trendline in (**B**) indicates that an association was found

**Fig. 4:**
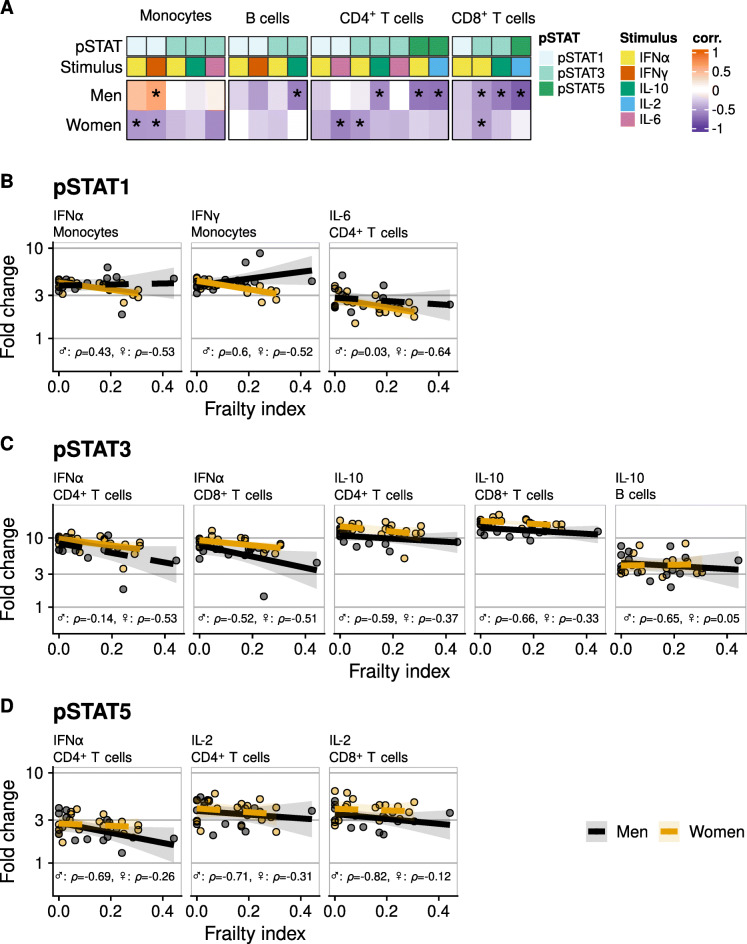
**A** heatmap showing relations between frailty and the cellular response to cytokines detected by phosphorylation of STAT1, STAT3, and STAT5 (fold change from baseline levels) in monocytes, B cells, CD4^+^ T cells and CD8^+^ T cells. Every box displays the Spearman’s *ρ* value, based on data of 16 men and 18 women. Scatterplots of conditions in which an association was detected for pSTAT1 (**B**), pSTAT3 (**C**) and pSTAT5 responses. For comparison, data of men and women are shown in the same plots. Solid trendlines indicate that an association was found, and dashed line indicates that no association was found

We related frailty score to baseline pSTAT levels and found a negative association between a higher frailty score and baseline pSTAT1 in monocytes of men but not women (Fig. 3A and B). When we investigated the relationship of frailty with the cellular pSTAT cytokine responsiveness (upon cytokine stimulation), most of the observed associations were negative, i.e. negative correlations, and differed between men and women (Fig. 4A).

Associations of pSTAT1 responsiveness with frailty were mainly seen in women (Fig. 4A, B). pSTAT1 responses of monocytes (Fig. 4B, *ρ* = − 0.53 after IFNα and *ρ* = − 0.52 after IFNγ stimulation) and of CD4^+^ T cells (Fig. 4B, *ρ* = − 0.64 after IL-6 stimulation) were found to be weaker in women with a higher frailty index score (‘frailer’ women). In contrast, pSTAT1 responses of monocytes were found to be stronger in frailer men (*ρ* = 0.60) after IFNγ stimulation. Negative associations of pSTAT3 responsiveness with frailty were seen in B cells of men and T cells of both men and women, with most associations observed in men (Fig. 4A, C). IFNα-induced pSTAT3 responses were negatively related to frailty in CD4^+^ T cells of women but not in men, and to CD8^+^ T cells of both men and women. In men, other negative relationships were found between IL-10-induced pSTAT3 responses and frailty in B cells, CD4^+^ T cells, and CD8^+^ T cells (*ρ* = − 0.65, *ρ* = − 0.59, *ρ* = − 0.66, respectively). Lastly, associations of pSTAT5 responsiveness with frailty were only seen in T cells of men (Fig. 4D). Negative associations in men were found of IFNα-induced pSTAT5 responses with frailty in CD4^+^ T cells (*ρ* = − 0.69) and of IL-2 induced pSTAT5 responses with frailty in CD4^+^ and CD8^+^ T cells (*ρ* = − 0.71 and − 0.82, respectively).

It is known that BMI can influence the inflammatory profile and that BMI is generally higher in frail individuals. When associations were directly tested between BMI and the cellular pSTAT responses, they had similar direction as with frailty, but none of the associations was strong enough with an acceptable false discovery rate (Fig. S7).

Thus, in general multiple associations of pSTAT responsiveness with frailty were seen, with overall lower cytokine induced cellular responsivity in frailer individuals. The direction of the associations was mostly similar in men and women. However, the strength of the associations differed, with more associations found in men. Furthermore, impaired pSTAT1 responsiveness of monocytes was found in frailer women and impaired pSTAT5 responsiveness of CD4^+^ and CD8^+^ T cells in frailer men.

### Defective regulatory pSTAT signaling in men with chronic low-grade inflammation

**Fig. 5:**
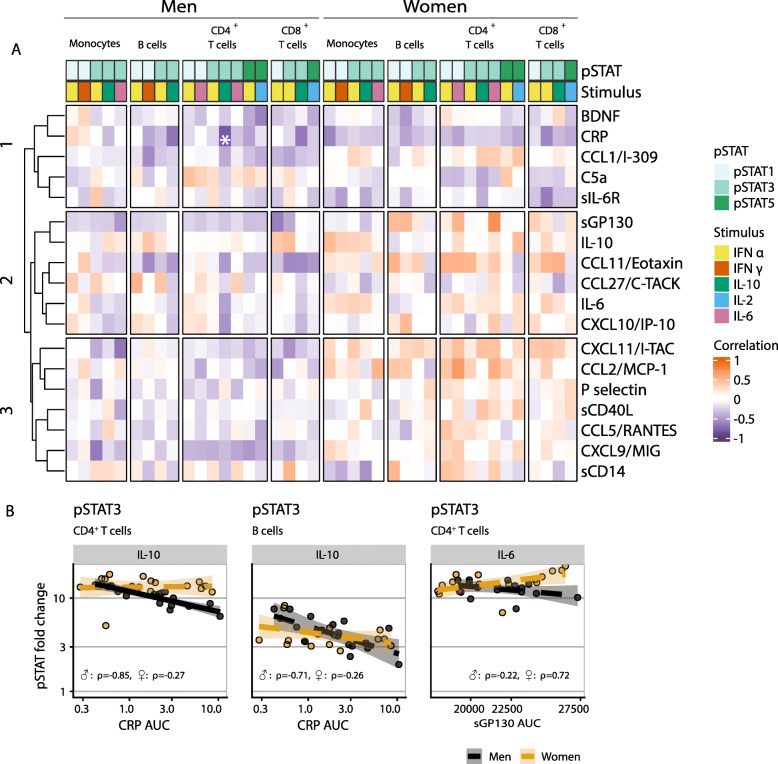
**A** heatmap showing Spearman’s correlation coefficients between immune cell pSTAT responses and systemic levels of inflammatory markers measured over the last 20 years (AUC). The magnitude and direction of the correlation are indicated by shades of color. Every correlation is based on *n* = 18 women or *n* = 16 men. A star indicates that an association was found. **B** Scatterplots showing relationships between immune cell pSTAT responses and systemic levels of inflammatory markers (AUC), of which Spearman’s *ρ* was > 0.7 or < − 0.7. For comparison, data of men and women are shown in the same plots. Solid trendlines indicate that an association was found, and dashed line indicates that no association was found

Since chronic low grade inflammation may impact cellular responsiveness, we next explored whether cellular pSTAT levels were related to any of 18 different inflammatory markers that were measured longitudinally, namely with 5-year intervals over the past 20 years in the same individuals. Cumulative exposure to low-grade inflammation over this time period was estimated, by calculating the area under the curve of every inflammatory marker for each individual. We then related the AUC’s to their cellular pSTAT responses. A clustering algorithm identified three clusters of markers based on Spearman’s *ρ* values (Fig. 5A). As the heatmap shows, positive correlations were more prominent in women, which contrasted with the more prominent negative ones in men, especially in clusters 2 and 3. Higher CRP levels in the past 20 years in men were associated with lower IL-10 induced pSTAT3 responses (Fig. 5A, B), which might indicate defective regulatory IL-10 signaling in men. This association was the one with the highest *ρ* value (*ρ* = − 0.85) and the only one below the pre-set false discovery rate threshold. Other high *ρ* values (*ρ* > 0.7 or *ρ* < − 0.7) were also seen between CRP levels and IL-10 induced pSTAT3 responses in B cells in men (*ρ* = − 0.71) (Fig. 5B). In women the highest *ρ* value was found for the relationship between sGP130 levels and IL-6 induced pSTAT3 CD4^+^ T cell responses (*ρ* = 0.72). Of note is that, while frailty was related to several pSTAT responses induced by IL-10 and IL-6, no association was found of pSTAT responsiveness with IL-10 or IL-6 levels in the circulation. Thus, only in men associations were found between pro-inflammatory marker levels in the past 20 years and pSTAT responses, possibly showing signs of a defective IL-10 signaling.

### No associations found between frailty and immune cell cytokine production after TLR stimulation

**Fig. 6:**
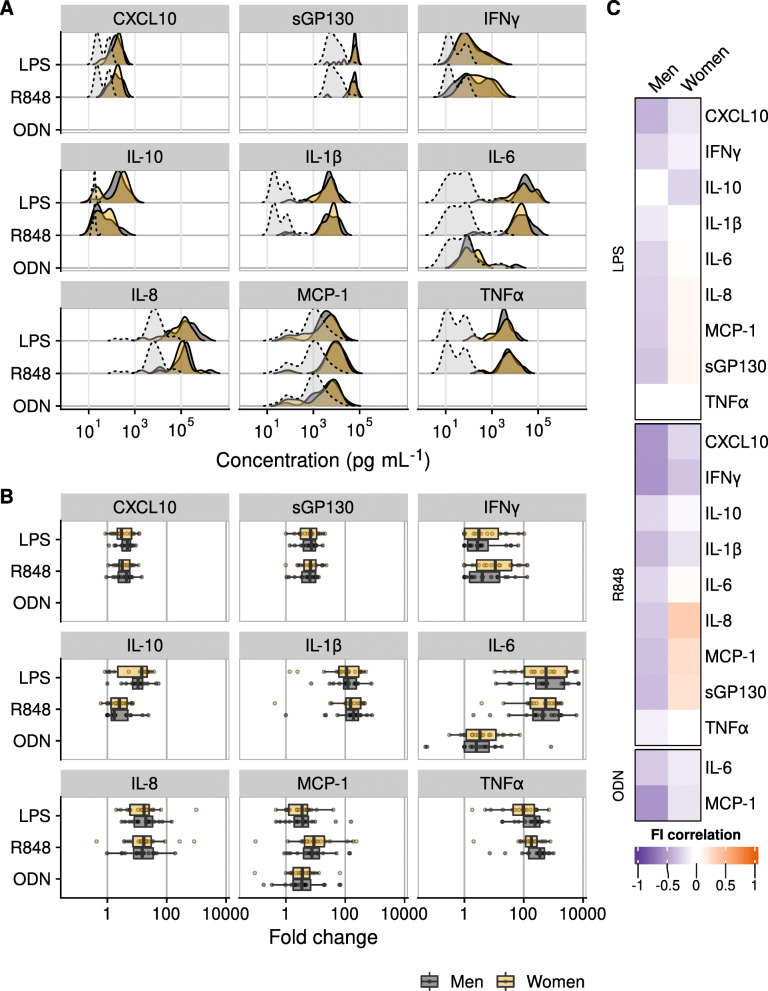
**A** Density plots of cytokine production by PBMCs after 24 h stimulation with medium (light grey density, spontaneous cytokine production) or with R848, ODN, or LPS. **B** Cytokine production by PBMCs upon 24 h stimulation with TLR4 (LPS), TLR7/8 (R848), and TLR9 (CpG ODN) agonists. Plots are shown only for cytokines with a fold change > 2. **C** Heatmap showing Spearman’s *ρ* values of correlations between frailty and PBMC cytokine production

We also investigated if cytokine production is different in frail individuals after activation of specific pattern recognition receptors that are thought to be involved in inflammation. After PBMCs were stimulated for 24 h with agonists of either Toll-like receptor (TLR)4 (lipopolysaccharide, LPS), TLR7/8 (Resiquimod, R848) or TLR9 (CpG ODN), we measured production of the cytokines IFNα, IFNγ, TNFα, IL-10, IL-1β, IL-8, MCP-1, CXCL10 and of sGP130. Stimulation with LPS and R848 resulted in enhanced cytokine production for most cytokines, while CpG ODN stimulation generally resulted in no or low cytokine production. Levels of IFNα were below detection limit after 24 h stimulation for the majority of the samples. A selection was made of 20 ‘clear’ responses to TLR-ligands (‘clear’ defined as median > 2-fold change in cytokine production compared to control sample), which were used for further analyses (Fig. 6A, B). Cytokine production after TLR activation of PBMCs did not differ between men and women when testing either cytokine concentrations after stimulation (Fig. 6A) or fold change in concentration (Fig. 6B, Table S9). Regarding correlations between frailty and PBMC cytokine production conditions, more negative Spearman’s *ρ* values were found in men than in women, but no association with frailty was found in either men or women (Fig. 6C, Table S10).

## Discussion

Our main finding is that frailty in older individuals of 65–74 years coincides with lower cellular pSTAT responsiveness. Negative associations between pSTAT responsiveness and frailty were found in both men and women. More and stronger associations were found in men, with signs of lower immune regulatory (IL-10 induced) pSTAT3 responses in frailer men. These lower responses in frailer men were related to higher levels of CRP in the past 20 years. Our results extend previous knowledge that showed lower pSTAT responses at higher age and in cardiovascular dysfunction [17], and underline the importance of the JAK-STAT pathway in frailty and, possibly, immunosenescence.

To our knowledge, no other studies related cellular pSTAT responsiveness to frailty. Lower pSTAT responses were previously found to be related to markers of cardiovascular disease and to chronological age [17]. More specifically, IFNα-induced pSTAT3 in CD8^+^ T cells and pSTAT5 in CD4^+^ T cells were negatively associated with age. Our data extend these findings by showing that IFNα-induced pSTAT3 responses of CD8^+^ T cells were also negatively associated with frailty in both men and women and that IFNα-induced pSTAT5 responses in CD4^+^ T cells were negatively associated with frailty in older men. While previous studies found higher levels of baseline pSTAT levels at higher ages [17, 18] and with higher CRP levels [17], we found an opposite relationship with frailty instead, namely lower baseline pSTAT1 levels in monocytes of frailer men. Given these previous findings, this was unexpected since chronic low-grade inflammation is seen more often in frail individuals [32–34]. Thus, it is unclear how this should be interpreted with regard to the theory that immune cells of frail older people show higher baseline JAK-STAT pathway activation. We speculate that baseline STAT phosphorylation is reduced in immune cells of frail elderly due to these cells being continuously triggered by chronic low-grade inflammation, leading to cellular exhaustion; further studies are needed to confirm this hypothesis. On the other hand, impaired cytokine signaling may cause reduced immune responses, leading to accumulation of damage-associated molecular patterns and thereby induction of chronic low-grade inflammation. Thus, while it is unclear whether chronic low-grade inflammation is a cause or a consequence of impaired cytokine signaling, both can enhance each other, are part of a dysfunctional immune system and probably lead to reduced immune responses that are clinically meaningful, due to their associations with frailty.

Of interest is the association we found between higher CRP levels in the previous 20 years, a sign of chronic low grade inflammation, and lower IL-10 induced pSTAT3 responses in men. Since we also observed lower pSTAT3 responsiveness to IL-10 in lymphocytes of frailer men, this might suggest that their lymphocytes are less responsive to anti-inflammatory signals and therefore less able to control inflammation. These findings are in agreement with results of previous studies showing that chronically elevated levels of CRP are related to frailty [32–34]. Recent studies noted that severely ill COVID-19 patients show elevated levels of IL-10 [35] which led some investigators to propose that IL-10 might contribute to the severity of the disease, because IL-10 is known to have both pro- and anti-inflammatory properties [36]. Given that the risk of developing severe symptoms in COVID-19 is greater in older men than in older women [37], and since we showed signs of defective IL-10 signaling in frail men, we speculate that defective downstream IL-10 -STAT3 signaling contributes to the severity of infectious diseases such as COVID-19 due to the reduced immune regulatory function of IL-10. Indeed, JAK-STAT pathway signaling has been shown to be pivotal in developing severe symptoms such as a cytokine release syndrome in COVID-19 patients [38], in particular through STAT3 signaling [39].

Many clinical trials are currently ongoing to investigate efficacy of blocking JAK-STAT signaling with JAK inhibitors in the treatment of COVID-19 [38]. While phase II studies showed promising results [40, 41], early reports from phase III trials are mixed, with some positive [42] and some negative results [43]. These mixed results might be explained by different timing of drug administration, and by improper identification of the patients who benefit most from JAK/STAT inhibition [38]. This should be addressed in future studies which should also elucidate whether impaired regulatory IL-10 STAT3 signaling can be a marker of developing severe reactions to infectious diseases. This would strengthen the rationale for targeting this pathway. Thus, cellular pSTAT levels may qualify as a biomarker to help to identify patients that benefit most from JAK inhibitor treatment.

An important factor in the association between frailty and reduced cytokine responsiveness could be overweight, since overweight has been associated with impaired JAK-STAT responses in adipocytes [44]. We were unable to adjust the results for BMI directly due to the small sample size. However, we did not find associations of pSTAT responses with BMI, which may imply that BMI is not the main driver in the association of pSTAT1, pSTAT3, and pSTAT5 responses in immune cells and frailty.

While we found that cytokine responsiveness is lower in frail people, we did not find an association of frailty with cellular cytokine production after stimulation with TLR agonists such as LPS, R848 and CpG ODN. This might mean that the initial intracellular reaction to pattern associated molecular patterns is still intact, but that the response to TLR-induced cytokines is reduced in frail individuals due to reduced JAK-STAT pathway signaling. Results should be interpreted with care, as the high dosage of the TLR agonists chosen for in vitro stimulation may overcome or mask possible subtle in-vivo differences in TLR responses.

To the best of our knowledge, this is the first study describing differences in pSTAT responses of leukocytes in men and women. Lower cellular pSTAT responsiveness in frailer participants was seen in both men and women, with the direction of the correlations being the same in men and women for most of the experimental conditions. However, impaired signaling in frailer participants was seen with different pSTATs in men and women, and in general the observed associations were stronger in men. Of interest is that pSTAT1 cytokine signaling was found to be more impaired in frail women than in men, while frail women were also found to have higher monocyte numbers. This is consistent with impaired phagocyte functioning in frail women, with more monocytes being produced due to their impaired cellular signaling capacity as a possible compensatory mechanism. On the other hand, pSTAT5 signaling was found to be impaired in frail men but not in frail women. It is known that pSTAT5 responses can be initiated by growth hormones [45]. Growth hormone secretion is higher in women than in men and is influenced by sex hormones such as estrogens [45], which might explain why we found the associations between pSTAT5 responses and frailty in men but not in women. Sex hormone levels might also further help to explain the differences in pSTAT responsiveness that we found between men and women, since STATs are known to interact with the androgen receptor [46] and pSTAT activation is influenced by estrogen and progesterone levels [47, 48].

Strengths of the study are the in-depth pSTAT signaling assay that we used, which allowed us to characterize a wide range of functional cellular cytokine response signals. Another advantage is that we could build on the extensive information and biomaterials gathered in the course of a unique longitudinal cohort study. This gave us the opportunity to compile and score a comprehensive frailty index in older individuals and to measure inflammatory markers longitudinally over a period of 20 years to quantify chronic low-grade inflammation. Our study also comes with limitations. First, our sample size was relatively low, so results cannot be extrapolated to the general population. Furthermore, the cellular pSTAT responsiveness could not accurately predict frailty by a prediction model due to the small sample size and the relatively small changes in effect size (data not shown). Future studies with larger populations, preferably including participants that are young and healthy both with and without low-grade inflammation, could help to establish clinical usefulness of our results and to determine whether defective signaling precedes frailty or not. In addition, it would be useful to measure levels of reproductive hormones to evaluate their influence on reduced pSTAT signaling in frail individuals. Another noteworthy point is that, while we used a large panel of cytokines signaling via JAK/STAT pathways we did not study activation of other known STATs, namely STAT2, STAT4 and STAT6. Larger phospho-flow panels may give a more complete description of cellular responsiveness in frail older people. Also, expansion of the staining panels with, for example, phenotypic markers to improve CD8^+^ T cell gating, could also be considered in future studies. Furthermore, other innate signaling cytokines such as IL-4 and IL-8 could be considered to be included, especially since the function of myeloid lineage cells is thought to become impaired with age [5, 49] and numbers of myeloid cells are higher in frail older people [31]. Lastly, how the results translate to in-vivo signaling remains to be investigated. In-vivo signaling might differ from in-vitro signaling, as the context is different and in vivo cytokine receptor expression might vary due to e.g. the presence of specific antigen, co-stimulation, or a difference in strength and duration of stimulation.

## Conclusion

In summary, our study gives important insights into relationships between immune functioning and frailty, and revealed sex-specific differences. We found cellular pSTAT responses to be reduced in older frail individuals. Interestingly, frail men show signs of defective regulatory pSTAT cytokine signaling and this is associated with chronic low-grade inflammation in the past 20 years. The data imply that the JAK/STAT signaling pathway is important in the aging process and therefore markers of this pathway could have utility as biomarkers of frailty. This is of particular interest since multiple drugs have been developed that can target the JAK/STAT pathway. We hope that these data encourage further research investigating how impaired JAK-STAT signaling is related to a poor immunological response to vaccines and infections and to chronic low-grade inflammation in older individuals.

## Methods

The study participants take part in the ongoing Doetinchem cohort study (DCS) [26, 27]. In the DCS, six consecutive measurement and sampling rounds, every 5 years, have been completed (1993–1997, 1998–2002, 2003–2007, 2008–2012, 2013–2017), while the seventh round is still ongoing (2018–2022). During each measurement round, plasma samples were taken and information regarding the participants’ health was collected. The present analysis is based on a sample of 16 men and 18 women who were 65–74 years of age, selected from a subgroup of DCS participants in which we previously analyzed inflammatory marker trajectories [50]. The latter were still active in the DCS at least until 2016, had at least 5 plasma samples available from previous rounds, were CMV seronegative at last measurement, and did not use immunosuppressants. From among those, we chose individuals with the highest and with the lowest frailty index, in equal numbers. The sample size was restricted by budgetary and logistic constraints.

### Frailty index

Frailty of participants was evaluated with a frailty index score. Details on the frailty index score used in this study can be found elsewhere [33]. In short, the frailty index score was based on previous studies [24, 25, 51, 52] and consists of 36 health deficits assessed by questionnaires and objective measurements. Examples are cognitive deficits obtained from validated cognitive tests, physical deficits such as poor handgrip strength, psychological deficits, and deficits regarding living independently (Table S2). The values that the frailty index can take are restricted between 0 (best possible score) and one (worst possible score). The frailty index was calculated based on data of the last two DCS assessment rounds (round 5 and 6) and was validated in the Doetinchem cohort study [33].

### PBMC isolation

Peripheral blood mononuclear cells (PBMCs) were isolated from heparinized blood by Lymphoprep (Progen) density gradient centrifugation, according to the manufacturer’s instructions. After isolation, the cells were washed with PBS (Gibco) containing 0.2% FCS, and then frozen in a solution with 90% fetal calf serum and 10% dimethyl sulfoxide at − 135 °C until further use.

### PBMC cytokine response analysis using phospho-flow cytometry

PBMCs from all participants (*n* = 34) were stimulated with cytokines to measure JAK-STAT pathway activation by quantifying the phosphorylation of STAT 1, 3 and 5 (Fig. 1). We used a barcoding technique as described previously [17, 53]. PBMCs were rapidly thawed and washed in RPMI (Thermofisher Scientific) with 10% fetal bovine serum. After 1 h resting, the PBMCs were stimulated with one cytokine per well (deep well plates, Sigma-Aldrich, 0.5*10^6^ PBMCs per well) for 30 min at 37 °C, 5% CO_2_ with the cytokines IL-2 (R&D Systems, 50 ng mL^− 1^), IL-6 (R&D Systems, 25 ng mL^− 1^), IL-10 (Peprotech, 100 ng mL^− 1^), IFNα (R&D Systems, 0.5*10^4^ U mL^− 1^) and IFNγ (Peprotech, 50 ng mL^− 1^). One well was reserved as the control condition, without stimulus. Next, PBMCs were fixated (in the deep well plate) with 1.6% paraformaldehyde (Alfa Aesar) for 10 min at room temperature, and thereafter permeabilizated with ice-cold (− 20 °C) 100% methanol for 5 min, at 4 °C. After permeabilization, the methanol was diluted 1:1 with cold PBS and the individual samples were stained for 30 min, 4 °C with a combination of Pacific Orange succinimidyl esters (0, 0.13, or 1 μg mL^− 1^) and Alexa Fluor 750 succinimidyl esters (0, 0.5, or 2 μg mL^− 1^ Fisher Scientific), to obtain a unique bar code per condition. After barcoding, the PBMCs were extensively washed with PBS/0,5% BSA/2 mM EDTA and the PBMCs from the same individual were collected and pooled in one FACS tube (BD Falcon). Thereafter, the PBMCs were stained for 30 min at 4 °C with CD3(UCHT1)-Pacific Blue, CD4(SK3)-BUV395, CD20(H1)-PerCPCy5.5, and CD33(P67–6)-PECy7 to distinguish CD4^+^ and CD8^+^ T cells, B cells, monocytes, respectively and with the markers STAT1(4a)-Alexa Fluor 488, STAT3(4/P)-Alexa Fluor 647 and STAT5(pY694)-PE to quantify phosphorylation of STATs (Fig. S2). Samples were measured on a flow cytometer (LSR II Fortessa X20, BD Bioscience).

### TLR stimulation of PBMC and cytokine production assay

PBMCs of all participants were thawed, washed and put to rest for 1 h in RPMI+glutamax with 10% FBS in a 96-wells round-bottom plate (Greiner), at a concentration of 200.000 PBMCs/well. After resting, the PBMCs were stimulated with one stimulus per well for 24 h at 37 °C, 5% CO_2_. Stimuli used were LPS EK (E.coli K12 ultrapure) 10 ng mL^− 1^ (TLR4), CpG ODNM362 10 μg mL^− 1^ (TLR9), and R848 10 μg mL^− 1^ (all Invivogen) (TLR7&8). After stimulation, the supernatants were collected and stored at − 80 °C for later use. The supernatants were thawed and analyzed in two batches on a FACSCanto™ flow cytometer (BD Biosciences), using a custom-made bead-based immunoassay (LEGENDplex™, BioLegend) according to the manufacturer’s instructions, for the following cytokines and chemokines: sGP130, IL-6, IFNγ, IL-10, IL-1β, TNFα, MCP-1, RANTES, CXCL8/IL-8, CXCL10/IP10, and IFNα. In addition, concentrations of sIL6R were quantified using a commercially available ELISA kit (R&D Systems).

### Plasma inflammatory protein trajectories

To quantify low-grade inflammation, we measured a panel of inflammatory markers in 5 repeated blood samples per individual that were withdrawn at 5-year intervals over the period of approximately 20 years; details are described elsewhere [50]. The panel consists of the following cytokines, chemokines and soluble receptors: C-C Motif Chemokine Ligand (CCL) 1/I-309, CCL2/MCP-1, CCL5/RANTES, CCL11/Eotaxin, CCL27/C-TACK, C-X-C Motif Chemokine Ligand (CXCL) 9 /MIG, CXCL10/IP-10, CXCL11/I-TAC, IL-10, IL-6, soluble CD40 ligand (sCD40L), soluble CD14 (sCD14), soluble IL-6 receptor (sIL-6R), glycoprotein 130 (GP130), Complement 5a (C5a), Brain-derived neurotrophic factor (BDNF), and soluble P-selectin. Plasma levels were measured in a multiplex immunoassay (Luminex core facility lab, UMC medical center, Utrecht, The Netherlands). Samples were stored at − 80^0^ C and only thawed on the morning of the measurement, with all samples from the same participant measured on the same plate. Levels of C-reactive protein (CRP) had been measured earlier in separate plasma samples and in a separate assay [54].

### Statistical analysis

#### Association studies

We used the permutation version of the Spearman’s test or, when appropriate, Wilcoxon’s test as implemented in the *coin* R package [55] to test associations for all our research questions. These association studies were performed separately for men and women. Associations were adjusted for possible confounding by batch effects. For all these association studies, we accounted for multiple testing by controlling the false discovery rate [56]. A cutoff of the false discovery rate of 15% was chosen based on the exploratory nature of this research. This means that, in theory, of all findings reported here at most 15% could be false positives. All details regarding the outcomes of the association studies, including *p*-values, are shown in supplementary information (Tables S2, S4, S5, S6, S7, S8, S9, and S10).

All analyses were performed in R (version 3.6.2) [57]. Several packages were used for general data wrangling [58, 59], data visualization [60–62], and customizing tables [63, 64]. Clusters of inflammatory markers within a heatmap were defined with a hierarchical cluster algorithm using the complete linkage criterion. The clustering was performed on the dissimilarity matrix which was obtained from data of the *ρ* values.

## Supplementary Information

**Additional file 1.**

## Data Availability

The datasets generated and/or analyzed during the current study are not publicly available due to privacy and ethical restrictions but are available from the corresponding author on reasonable request.
