# Supplementary material for: Impaired JAK-STAT pathway signaling in leukocytes of the frail elderly
Source: Immun Ageing. 2022 Jan 17;19:5. doi: 10.1186/s12979-021-00261-w (PMC8762193; doi:10.1186/s12979-021-00261-w)
Supplement: Supplementary file 1 — Additional file 1. [file 12979_2021_261_MOESM1_ESM.pdf]

Impaired JAK-STAT pathway signaling in leukocytes of frail elderly

Supplementary material

16 Dec 2021

Leonard Daniël Samson[1,2], Peter Engelfriet[1], W. M. Monique Verschuren[1,3], H. Susan J. Picavet[1],José A. Ferreira[1], Mary-lène de Zeeuw-Brouwer[1], Anne-Marie Buisman[1], A. Mieke H. Boots[2]

[1]: *National Institute of Public Health and the Environment, Bilthoven, The Netherlands*
[2]: *Department of Rheumatology and Clinical Immunology, University of Groningen, University Medical Center Groningen, The Netherlands*
[3]: *Julius Center for Health Sciences and Primary Care, University Medical Center Utrecht, Utrecht University, Utrecht, The Netherlands*

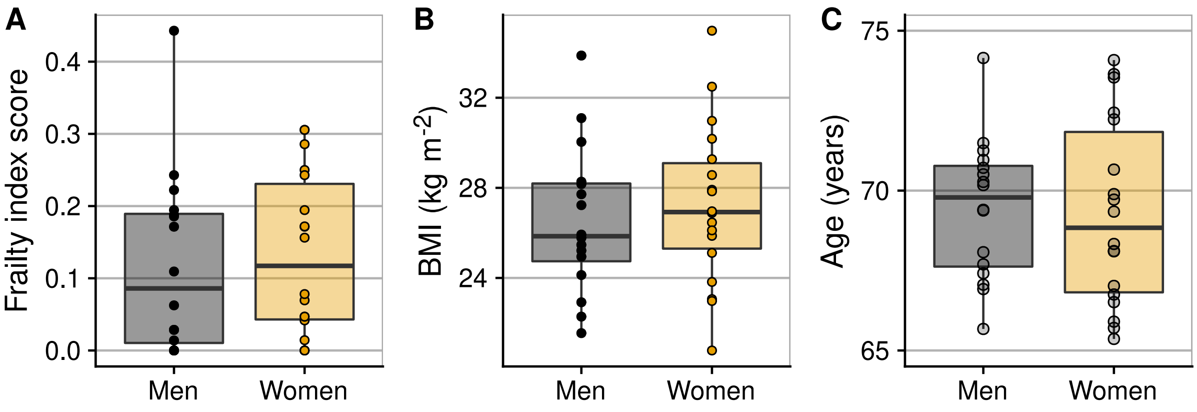

Figure S1: Frailty index score (A) BMI values (B) and age (C) in men and women in the study population (n=16 men, n=18 women).

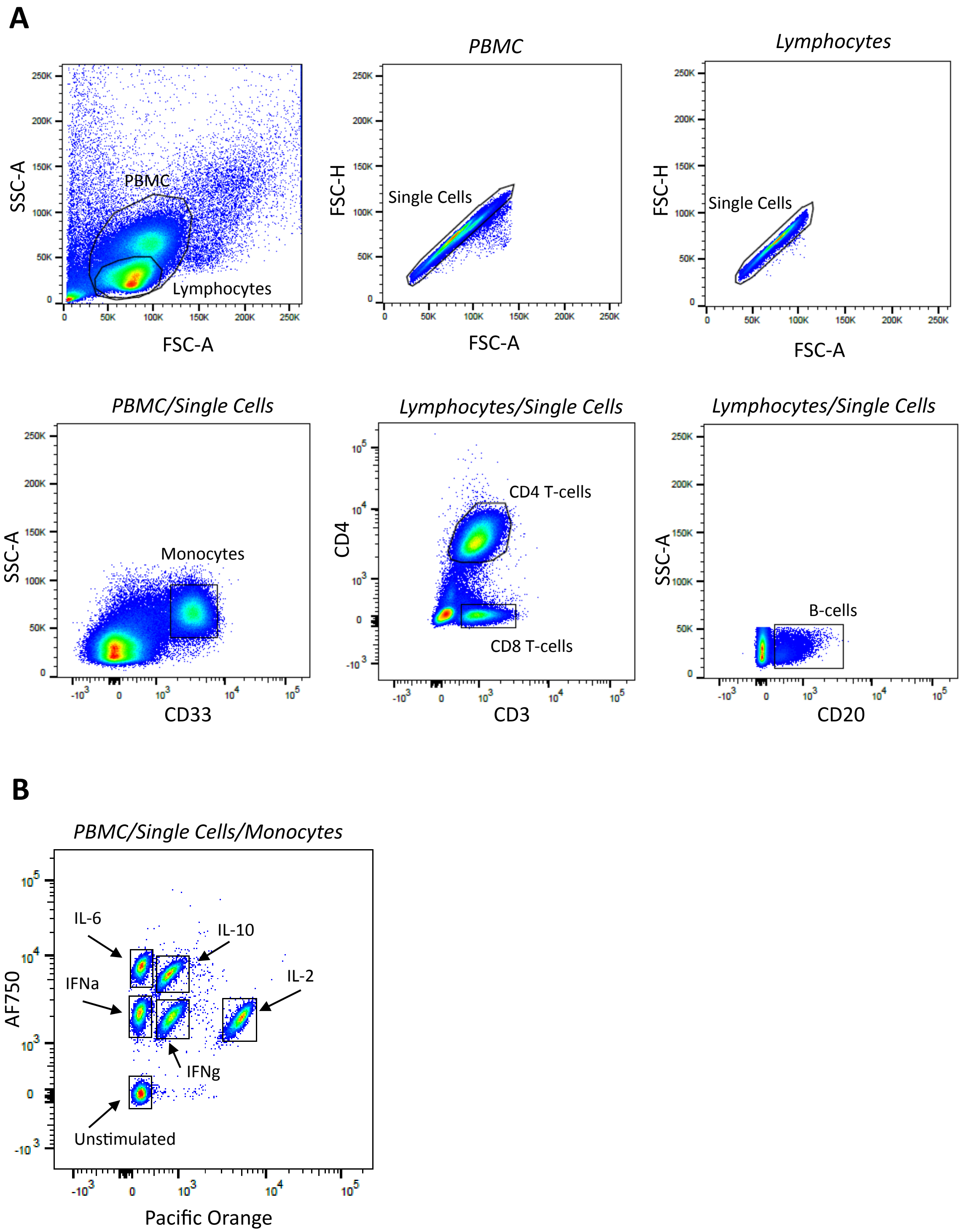

Figure S2: (A) Phospho-flow cytometry gating strategy, showing gating of leukocytes and lymphocytes, single cells, monocytes, B cells, $CD4^+$ T cells and $CD8^+$ T cells. (B) Gating strategy to separate different stimulus conditions after barcoding and pooling samples. Gating shown is within monocytes and is representative for the strategy within the other investigated cell subsets.

STAT1 expression in monocytes by IFNɣ stimulation - Women

STAT3 expression in CD8Tcells by IFNα stimulation - Men

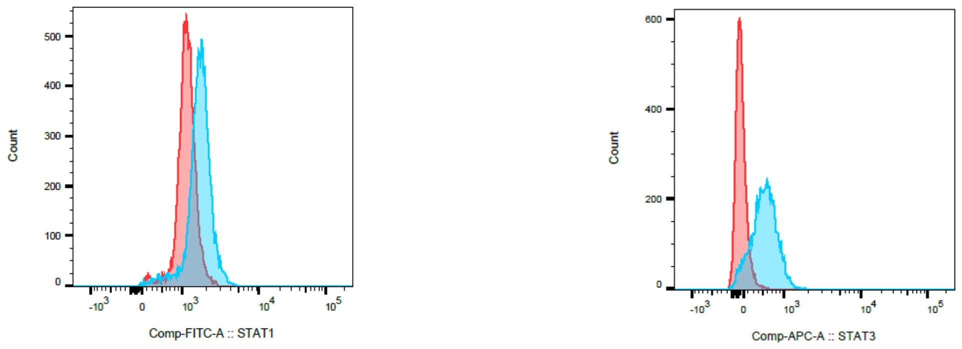

Blue: 'healthy' participant (FI: 0.00)
Red: 'frail' participant (FI: 0.31)

Blue: 'healthy' participant (FI: 0.00)
Red: 'frail' participant (FI: 0.24)

Figure S3: Examples of individual pSTAT expression histograms. Shown are histograms of IFNγ-induced pSTAT1 expression in monocytes (left figure) and IFNα-induced STAT3 expression in CD8$^+$ T cells (right figure). Histograms in both figures are from two representative women (left figure) or men (right figure) with either a low frailty index score (turquoise histograms) or high frailty index score (red histograms).

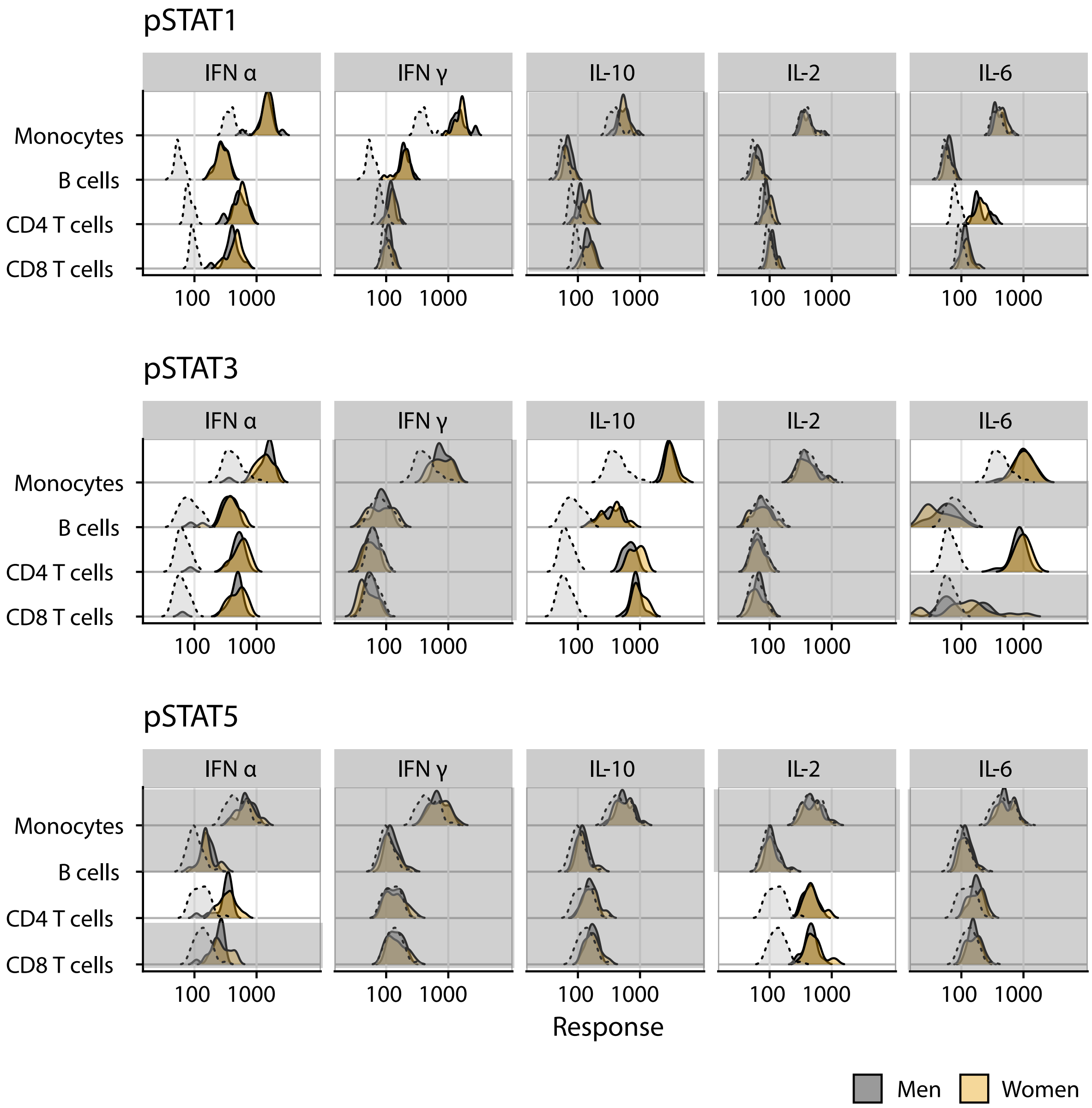

Figure S4: Phosphorylated STAT (pSTAT1, pSTAT3, pSTAT5) expression at baseline (unstimulated, light-grey densities with dashed lines) and after stimulation in men (dark grey) and women (yellow) with IFNα, IFNγ, IL-10, IL-2 or IL-6, in monocytes, B cells, CD4^{+} T cells, CD8^{+} T cells. Selected conditions for further analysis (median fold change>2) are shown with a white background, other plots (non-selected conditions) are presented with a dark grey background.

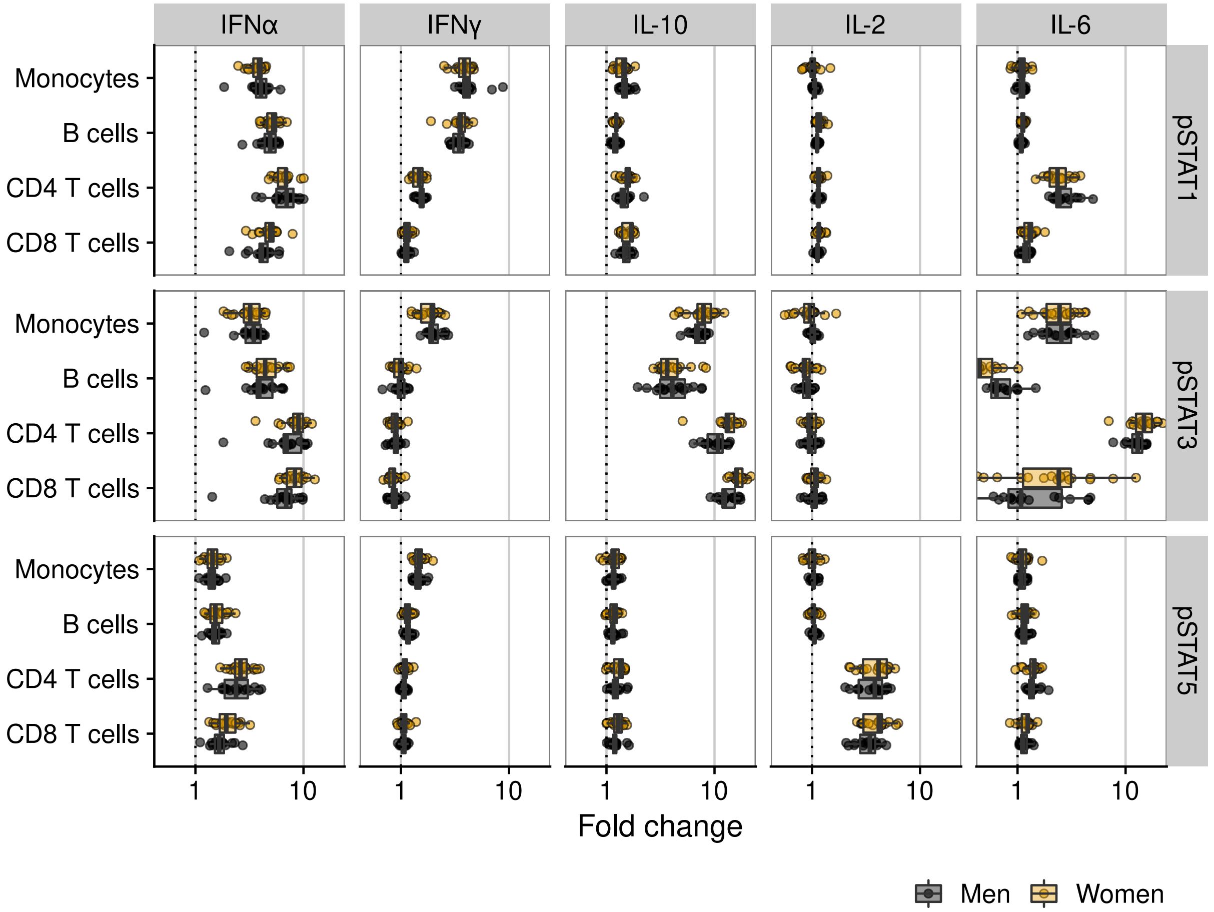

Figure S5: Fold change (stimulus/baseline) in pSTAT levels (pSTAT1, pSTAT3, pSTAT5) after stimulation with IFNα, IFNγ, IL-10, IL-2 or IL-6, in CD4$^+$ T cells, CD8$^+$ T cells, Monocytes, and B cells in men and women.

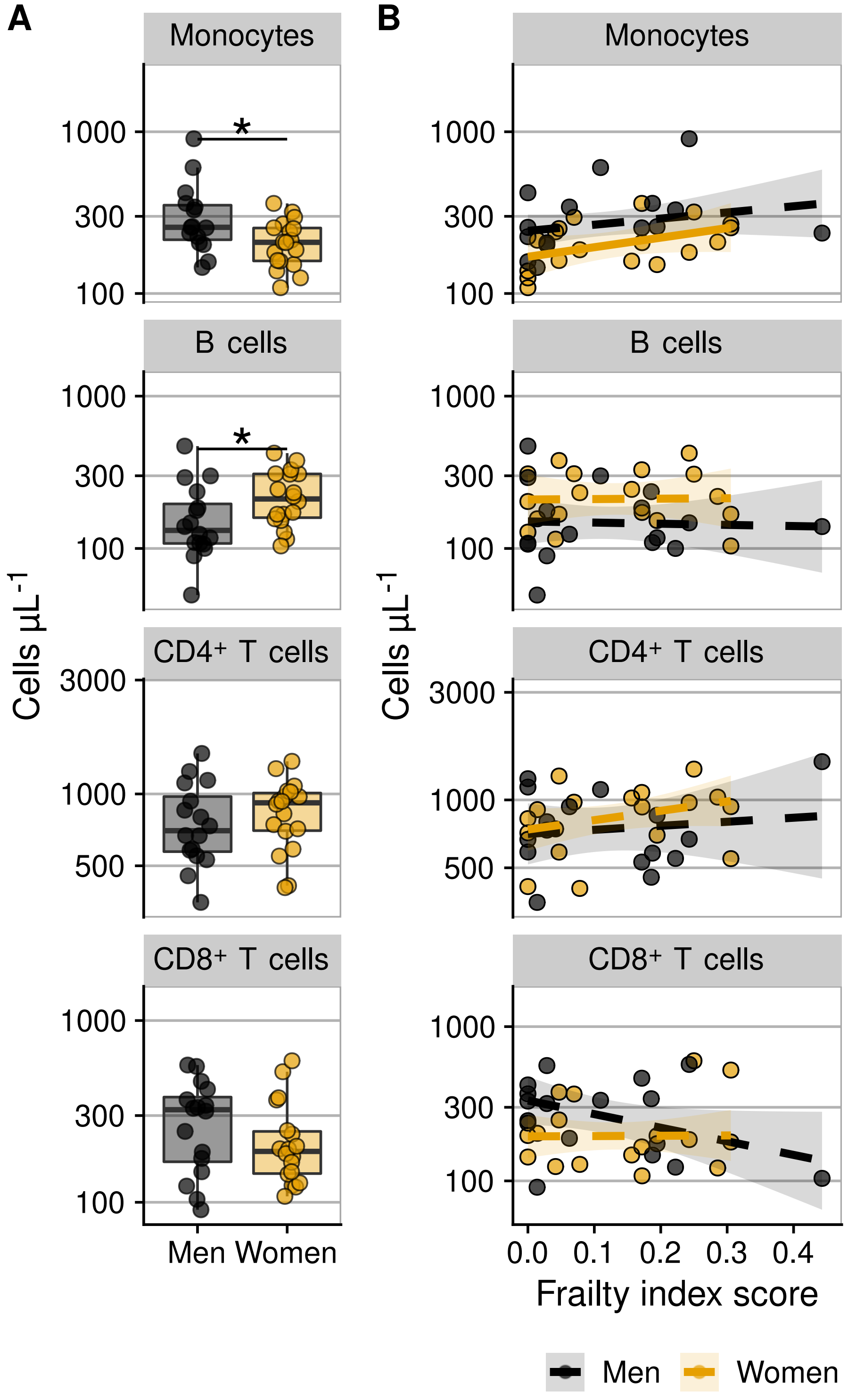

Figure S6: (A) Association between sex and immune cell numbers. *= association was found with false discovery rate <15%. (B) Association between immune cell numbers and frailty. Continuous trend line means association was found (with false discovery rate <15%), dashed line means that no association was found. Men: n=16, women n=18.

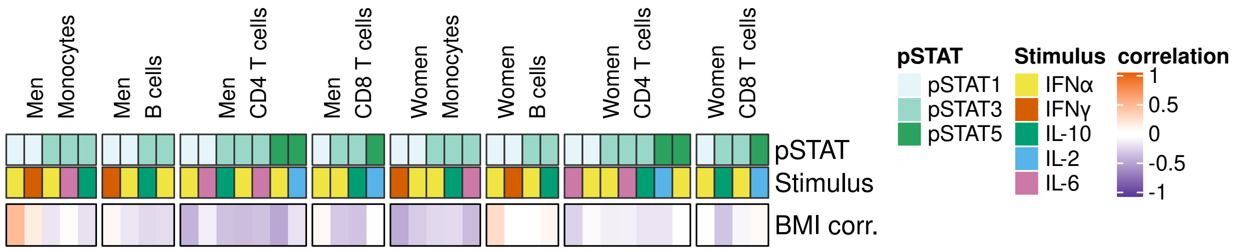

Figure S7: Heatmap showing the relation between BMI and cellular response to cytokines detected by phosphorylation of STAT1, STAT3, and STAT5 (fold change with baseline levels) in monocytes, B cells, $CD4^+$ T cells and $CD8^+$ T cells of men and women. Every box displays the Spearman's ρ value, based on n=18 women and n=16 men.

Table S1: Frailty index components

No	Frailty index component	Description	Value: 0	Value: 0.5	Value: 1
1	RR	High (systolic) blood pressure	$RR < 160$		$RR \geq 160$
2	Cardiologic Disease	One or more of the following conditions: myocardial infarction, bypass-surgery, balloon dilatation, cardiac catheterization, pacemaker implantation, large blood vessel surgery, hospitalization due to cardiac failure	No		One or more prevalent
3	Diabetes	Prevalence of Diabetes	No		Yes
4	Hearing	Inability to maintain a conversation in a group of 3 or more people due to hearing impairment (with hearing aid if needed)	Yes or with some effort		No or with great effort
5	Malignancy	(History of) any form of malignancy	No		Yes
6	Joint Inflammation	Chronic joint inflammation in the past year	No		Yes
7	Osteoporosis	Osteoporosis diagnosed by a medical doctor, in the past year,	No		Yes
8	Lower Back Pain	Severe lower back complaints in the past year (including lumbar herniated nucleus pulposus)	No		Yes
9	CVA	(History of) stroke	No		Yes
10	Migraine	Migraine prevalence in the past year	No		Yes
11	Neurologic Disease	One or more of the following neurological diseases, diagnosed by a medical doctor: m. Parkinson, Multiple Sclerosis, epilepsy	No		Yes
12	Asthma	Asthma diagnosed by a doctor and one or more asthma attacks in the past year	No		Yes
13	Spirometry Ratio	Poor lung function quantified by spirometry measurements: first second of forced expiration divided by the forced vital capacity (FEV1/FVC)	$FEV1/FVC > 0.70$		$FEV1/FVC \leq 0.70$
14	Digestive Tract	Severe bowel disorders in the past year, diagnosed by a medical doctor	No		Yes
15	Vertigo	Vertigo with falling the past 12 months	No vertigo	Some vertigo	Severe vertigo
16	BMI	Over- or underweight	$18.5 < BMI \leq 30$		$BMI \leq 18.5 or BMI > 30$

Table S1: Frailty index components *(continued)*

No	Frailty index component	Description	Value: 0	Value: 0.5	Value: 1
17	Pain	Limited in daily activities due to pain	No limitation	Some limitation	Severe limitation
18	Incontinence	Unintentional urine incontinence past 12 months	No		Yes
19	Ankle Brachial Index	Poor ankle brachial index (ABI, the ratio of the systolic blood pressure in the ankle to the blood pressure in the arm)	$ABI > 0.9$		$ABI \leq 0.9$
20	Health Perception	(Subjective) perception of poor health	No or some impairment		Severe impairment
21	Eyesight	Bad eyesight perception, facial recognition within 4 meters (with glasses/ contact lenses if needed)	No		Yes
22	Renal Function	Poor renal function (estimated Glomular Filtration Rate (eGFR), calculated with plasma creatinine concentrations) (Inker et al., 2012)	$eGFR \geq 60$		$eGFR < 60$
23	Cognitive Speed	Poor cognitive speed. Z-scores corrected for measurements per person and for education level. Scores derived from the Stroop Color-Word Test and the Letter-Digit Substitution Test, as described previously (Nooyens et al., 2011)	Not beloging to 10% participants with lowest z-score within the Doetinchem cohort		Belonging to 10% participants with lowest z-score within the Doetinchem cohort
24	Cognitive Memory	Poor cognitive memory. Z-scores corrected for measurements per person and for education level. Scores derived from the Verbal Learning Test, as described previously (Nooyens et al., 2011)	Not beloging to 10% participants with lowest z-score within the Doetinchem cohort		Belonging to 10% participants with lowest z-score within the Doetinchem cohort
25	Cognitive Flexibility	Poor cognitive flexibility. Z-scores corrected for measurements per person and education level. Scores derived from the Stroop Color-Word Test, as described previously (Nooyens et al., 2011)	Not beloging to 10% participants with lowest z-score within the Doetinchem cohort		Belonging to 10% participants with lowest z-score within the Doetinchem cohort
26	Physical Inactive	Not meeting the Dutch healthy exercise norm.(Kemper, 2000). In addition: belonging to the 25th lowest percentile of walking activity and the 10th percentile lowest low/medium/high intensive activities in the Doetinchem cohort	No		Yes

Table S1: Frailty index components *(continued)*

No	Frailty index component	Description	Value: 0	Value: 0.5	Value: 1
27	ADL	Limited in washing and dressing due to health	No limitation	Some limitation	Severe limitation
28	Household	Limited in daily activities (cooking, cleaning) due to health	No limitatlion	Some limitation	Severe limitation
29	Walking	Limited in in walking 100 meters	No limitatlion	Some limitation	Severe limitation
30	Lifting	Limited in lifting or carrying groceries due to health	No limitatlion	Some limitation	Severe limitation
31	Walking Stairs	Limited in climbing stairs	No limitatlion	Some limitation	Severe limitation
32	Grip Strength	Poor grip strength (cutoff points as described previously) (Fried et al., 2001)			
		$Men, BMI \leq 24$	> 29		≤ 29
		$Men, 24 < BMI \leq 26$	> 30		≤ 30
		$Men, 26 < BMI \leq 28$	> 30		≤ 30
		$Men, BMI > 28$	> 32		≤ 32
		$Women, BMI \leq 24$	> 17		≤ 17
		$Women, 23 < BMI \leq 26$	> 17.3		≤ 17.3
		$Women, 26 < BMI \leq 29$	> 18		≤ 18
		$Women, BMI > 29$	> 21		≤ 21
33	Depressed	Feeling depressed the past week	No limitatlion	Some limitation	Severe limitation
34	Happiness	Feeling unhappy the past 4 weeks	No limitatlion	Some limitation	Severe limitation

Table S1: Frailty index components *(continued)*

No	Frailty index component	Description	Value: 0	Value: 0.5	Value: 1
35	Mental Effort	Feeling as if every activity costs effort during the past week	No limitatlion	Some limitation	Severe limitation
36	Getting Going	Not being able to get going the past week	No limitatlion	Some limitation	Severe limitation

Table S2: Unstimulated pSTAT levels compared between men and women

Unstimulated condition	n	Direction	P value	FDR	Selected
pSTAT1-B cells	34	1	0.02	0.16	–
pSTAT3-CD8 T cells	34	1	0.03	0.17	–
pSTAT1-Monocytes	34	-1	0.11	0.35	–
pSTAT1-CD4 T cells	34	-1	0.14	0.35	–
pSTAT1-CD8 T cells	34	1	0.23	0.45	–
pSTAT5-CD8 T cells	34	1	0.38	0.63	–
pSTAT3-B cells	34	1	0.60	0.86	–
pSTAT3-Monocytes	34	1	0.61	0.77	–
pSTAT3-CD4 T cells	34	1	0.72	0.80	–
pSTAT5-CD4 T cells	34	1	0.80	0.80	–

Note:
Permutation version of the Wilcoxon-Mann-Whitney test was used. Tests are stratified by the 3 batches in which the phosflow measurements took place. FDR: (estimated) False Discovery Rate

Table S3: Selected phosflow conditions with fold change > 2

		Fold change		
Stimulus	pSTAT	all (n=34)	Men (n=16)	Women (n=18)
**Monocytes**				
IFNα	pSTAT1	3.9	4.0	3.9
IFNγ	pSTAT1	3.9	3.9	3.8
IFNα	pSTAT3	3.4	3.4	3.2
IL-10	pSTAT3	7.4	7.0	7.9
IL-6	pSTAT3	2.3	2.2	2.5
**B cells**				
IFNα	pSTAT1	4.9	4.9	5.2
IFNγ	pSTAT1	3.5	3.3	3.6
IFNα	pSTAT3	4.1	3.9	4.4
IL-10	pSTAT3	3.6	3.7	3.6
**CD4 T cells**				
IFNα	pSTAT1	6.8	6.7	6.9
IL-6	pSTAT1	2.5	2.5	2.3
IFNα	pSTAT3	8.1	7.4	9.0
IL-10	pSTAT3	11.9	10.7	13.4
IL-6	pSTAT3	13.9	12.9	14.8
IFNα	pSTAT5	2.5	2.4	2.6
IL-2	pSTAT5	3.8	3.7	4.1
**CD8 T cells**				
IFNα	pSTAT1	4.6	4.1	4.9
IFNα	pSTAT3	7.3	6.9	8.3
IL-10	pSTAT3	15.2	12.6	16.0
IL-2	pSTAT5	3.6	3.2	4.1

Table S4: Fold change in pSTAT levels compared between men and women

Stimulated condition	n	Direction	P value	FDR	Selected
IL-10-pSTAT3-CD8 T cells	34	-1	0.00	0.01	Yes
IL-10-pSTAT3-CD4 T cells	34	-1	0.01	0.12	Yes
IFNα-pSTAT1-CD8 T cells	34	-1	0.03	0.17	–
IFNα-pSTAT3-CD8 T cells	34	-1	0.03	0.16	–
IL-6-pSTAT1-CD4 T cells	34	1	0.04	0.17	–
IL-2-pSTAT5-CD8 T cells	34	-1	0.05	0.15	–
IFNα-pSTAT1-Monocytes	34	1	0.06	0.18	–
IFNα-pSTAT1-CD4 T cells	34	1	0.09	0.22	–
IFNα-pSTAT3-Monocytes	34	1	0.10	0.22	–
IFNγ-pSTAT1-Monocytes	34	1	0.13	0.25	–
IL-2-pSTAT5-CD4 T cells	34	-1	0.21	0.38	–
IL-6-pSTAT3-CD4 T cells	34	-1	0.27	0.44	–
IL-6-pSTAT3-Monocytes	34	1	0.32	0.49	–
IFNγ-pSTAT1-B cells	34	-1	0.42	0.60	–
IL-10-pSTAT3-Monocytes	34	-1	0.47	0.62	–
IFNα-pSTAT5-CD4 T cells	34	-1	0.48	0.61	–
IFNα-pSTAT1-B cells	34	-1	0.59	0.70	–
IFNα-pSTAT3-CD4 T cells	34	-1	0.84	0.94	–
IFNα-pSTAT3-B cells	34	1	0.94	0.99	–
IL-10-pSTAT3-B cells	34	1	0.98	0.98	–

Note:
Permutation version of the Wilcoxon-Mann-Whitney test was used. Tests are stratified by the 3 batches in which the phosflow measurements took place. FDR: (estimated) False Discovery Rate

Table S5: Unstimulated pSTAT levels compared with frailty in men and women

Sex	Stimulated condition	n	Direction	P value	rho	FDR	Selected
Men	pSTAT1-Monocytes	16	-1	0.01	-0.71	0.07	Yes
Men	pSTAT3-Monocytes	16	-1	0.13	-0.50	0.66	–
Men	pSTAT3-B cells	16	-1	0.28	-0.26	0.94	–
Men	pSTAT3-CD4 T cells	16	-1	0.36	-0.29	0.91	–
Men	pSTAT1-B cells	16	1	0.53	0.17	1.06	–
Men	pSTAT1-CD4 T cells	16	-1	0.66	-0.23	1.11	–
Men	pSTAT5-CD4 T cells	16	-1	0.80	-0.07	1.14	–
Men	pSTAT3-CD8 T cells	16	-1	0.85	-0.02	1.06	–
Men	pSTAT1-CD8 T cells	16	1	0.89	0.02	0.98	–
Men	pSTAT5-CD8 T cells	16	1	0.95	0.03	0.95	–
Women	pSTAT3-CD4 T cells	18	1	0.03	0.54	0.29	–
Women	pSTAT3-CD8 T cells	18	1	0.06	0.45	0.32	–
Women	pSTAT3-B cells	18	1	0.08	0.54	0.26	–
Women	pSTAT1-Monocytes	18	1	0.25	0.31	0.61	–
Women	pSTAT1-CD4 T cells	18	1	0.27	0.22	0.54	–
Women	pSTAT3-Monocytes	18	1	0.35	0.29	0.59	–
Women	pSTAT1-B cells	18	1	0.40	0.21	0.58	–
Women	pSTAT1-CD8 T cells	18	1	0.42	0.19	0.52	–
Women	pSTAT5-CD4 T cells	18	1	0.81	0.06	0.90	–
Women	pSTAT5-CD8 T cells	18	1	0.85	0.03	0.85	–

Note:
Permutation version of the Spearman test was used. Tests are stratified by the 3 batches in which the phosflow measurements took place. FDR: (estimated) False Discovery Rate.

Table S6: Associations of fold change in pSTAT levels with frailty in men and women

Sex	Stimulated condition	n	P value	rho	FDR	Selected
Women	IFNγ-pSTAT1-Monocytes	18	0.00	-0.52	0.09	Yes
Women	IFNα-pSTAT1-Monocytes	18	0.01	-0.53	0.09	Yes
Women	IL-6-pSTAT1-CD4 T cells	18	0.01	-0.64	0.08	Yes
Women	IFNα-pSTAT3-CD4 T cells	18	0.02	-0.53	0.11	Yes
Women	IFNα-pSTAT3-CD8 T cells	18	0.03	-0.51	0.12	Yes
Women	IL-6-pSTAT3-CD4 T cells	18	0.05	-0.40	0.18	–
Women	IL-6-pSTAT3-Monocytes	18	0.08	-0.59	0.23	–
Women	IFNα-pSTAT3-Monocytes	18	0.09	-0.36	0.23	–
Women	IL-10-pSTAT3-CD8 T cells	18	0.10	-0.33	0.22	–
Women	IFNα-pSTAT1-CD4 T cells	18	0.21	-0.41	0.41	–
Women	IL-10-pSTAT3-CD4 T cells	18	0.23	-0.37	0.41	–
Women	IFNα-pSTAT1-CD8 T cells	18	0.25	-0.29	0.41	–
Women	IL-10-pSTAT3-Monocytes	18	0.25	-0.29	0.39	–
Women	IFNα-pSTAT3-B cells	18	0.36	-0.31	0.52	–
Women	IL-2-pSTAT5-CD4 T cells	18	0.49	-0.31	0.65	–
Women	IFNα-pSTAT5-CD4 T cells	18	0.55	-0.26	0.68	–
Women	IL-10-pSTAT3-B cells	18	0.61	0.05	0.72	–
Women	IFNα-pSTAT1-B cells	18	0.62	-0.05	0.69	–
Women	IFNγ-pSTAT1-B cells	18	0.81	-0.27	0.85	–
Women	IL-2-pSTAT5-CD8 T cells	18	0.98	-0.12	0.98	–
Men	IL-2-pSTAT5-CD8 T cells	16	0.00	-0.82	0.01	Yes
Men	IL-2-pSTAT5-CD4 T cells	16	0.00	-0.71	0.00	Yes
Men	IFNα-pSTAT5-CD4 T cells	16	0.00	-0.69	0.01	Yes
Men	IFNα-pSTAT3-CD8 T cells	16	0.01	-0.52	0.05	Yes
Men	IL-10-pSTAT3-B cells	16	0.02	-0.65	0.08	Yes
Men	IL-10-pSTAT3-CD4 T cells	16	0.03	-0.59	0.09	Yes
Men	IL-10-pSTAT3-CD8 T cells	16	0.04	-0.66	0.13	Yes
Men	IFNγ-pSTAT1-Monocytes	16	0.06	0.60	0.15	Yes
Men	IFNα-pSTAT1-CD4 T cells	16	0.07	-0.30	0.17	–
Men	IFNα-pSTAT1-CD8 T cells	16	0.11	-0.31	0.21	–
Men	IFNγ-pSTAT1-B cells	16	0.11	-0.51	0.21	–
Men	IFNα-pSTAT3-CD4 T cells	16	0.20	-0.14	0.34	–
Men	IFNα-pSTAT1-B cells	16	0.22	-0.26	0.33	–
Men	IFNα-pSTAT3-B cells	16	0.23	-0.20	0.33	–
Men	IFNα-pSTAT1-Monocytes	16	0.35	0.43	0.47	–
Men	IFNα-pSTAT3-Monocytes	16	0.50	0.02	0.62	–
Men	IL-6-pSTAT3-Monocytes	16	0.59	0.13	0.70	–
Men	IL-6-pSTAT3-CD4 T cells	16	0.78	-0.02	0.86	–
Men	IL-10-pSTAT3-Monocytes	16	0.84	-0.15	0.88	–
Men	IL-6-pSTAT1-CD4 T cells	16	0.85	0.03	0.85	–

Note:
Permutation version of the Spearman test was used. Tests are stratified by the 3 batches in which the phosflow measurements took place. FDR: (estimated) False Discovery Rate.

Table S7: Associations of fold change in pSTAT levels with BMI in men and women

Sex	Stimulated condition	n	P value	rho	FDR	Selected
Women	IFNα-pSTAT3-Monocytes	18	0.07	-0.23	1.46	–
Women	IFNγ-pSTAT1-Monocytes	18	0.08	-0.44	0.84	–
Women	IFNα-pSTAT1-Monocytes	18	0.11	-0.29	0.75	–
Women	IFNα-pSTAT1-B cells	18	0.13	0.26	0.64	–
Women	IL-6-pSTAT1-CD4 T cells	18	0.16	-0.26	0.63	–
Women	IL-10-pSTAT3-CD8 T cells	18	0.20	-0.32	0.68	–
Women	IL-10-pSTAT3-Monocytes	18	0.26	-0.22	0.76	–
Women	IL-6-pSTAT3-Monocytes	18	0.27	-0.37	0.68	–
Women	IFNα-pSTAT3-CD4 T cells	18	0.46	-0.12	1.03	–
Women	IL-2-pSTAT5-CD4 T cells	18	0.46	-0.17	0.93	–
Women	IL-6-pSTAT3-CD4 T cells	18	0.48	-0.12	0.88	–
Women	IL-10-pSTAT3-CD4 T cells	18	0.53	-0.17	0.89	–
Women	IFNα-pSTAT5-CD4 T cells	18	0.68	-0.05	1.04	–
Women	IFNα-pSTAT3-CD8 T cells	18	0.71	-0.09	1.01	–
Women	IFNγ-pSTAT1-B cells	18	0.78	-0.01	1.04	–
Women	IFNα-pSTAT3-B cells	18	0.80	0.02	0.99	–
Women	IFNα-pSTAT1-CD8 T cells	18	0.87	0.04	1.02	–
Women	IFNα-pSTAT1-CD4 T cells	18	0.90	-0.08	1.00	–
Women	IL-2-pSTAT5-CD8 T cells	18	0.97	0.09	1.02	–
Women	IL-10-pSTAT3-B cells	18	0.99	0.10	0.99	–
Men	IFNα-pSTAT1-Monocytes	16	0.06	0.46	1.12	–
Men	IL-10-pSTAT3-CD4 T cells	16	0.06	-0.34	0.63	–
Men	IFNα-pSTAT1-CD4 T cells	16	0.10	-0.41	0.65	–
Men	IFNα-pSTAT3-CD8 T cells	16	0.15	-0.30	0.74	–
Men	IL-10-pSTAT3-CD8 T cells	16	0.15	-0.33	0.62	–
Men	IFNα-pSTAT5-CD4 T cells	16	0.17	-0.46	0.57	–
Men	IFNα-pSTAT3-CD4 T cells	16	0.22	-0.35	0.62	–
Men	IL-10-pSTAT3-B cells	16	0.27	-0.22	0.68	–
Men	IFNγ-pSTAT1-Monocytes	16	0.41	0.16	0.91	–
Men	IL-6-pSTAT1-CD4 T cells	16	0.49	-0.13	0.97	–
Men	IL-2-pSTAT5-CD4 T cells	16	0.50	-0.18	0.91	–
Men	IFNα-pSTAT3-Monocytes	16	0.50	-0.17	0.84	–
Men	IL-6-pSTAT3-Monocytes	16	0.55	0.06	0.85	–
Men	IL-6-pSTAT3-CD4 T cells	16	0.58	-0.33	0.83	–
Men	IL-10-pSTAT3-Monocytes	16	0.65	-0.19	0.87	–
Men	IFNγ-pSTAT1-B cells	16	0.66	0.10	0.82	–
Men	IFNα-pSTAT3-B cells	16	0.70	-0.21	0.83	–
Men	IFNα-pSTAT1-CD8 T cells	16	0.76	0.09	0.85	–
Men	IL-2-pSTAT5-CD8 T cells	16	0.86	0.01	0.91	–
Men	IFNα-pSTAT1-B cells	16	0.97	-0.16	0.97	–

Note:
Permutation version of the Spearman test was used. Tests are stratified by the 3 batches in which the phosflow measurements took place. FDR: (estimated) False Discovery Rate.

Table S8: Associations of fold change in pSTAT levels with the area under the curve of inflammatory markers in men and women

Sex	Stimulated condition	Marker	n	P value	rho	FDR	Selected
Men	IL-10-pSTAT3-CD4 T cells	CRP	16	0.00	-0.85	0.03	Yes
Men	IFNα-pSTAT3-CD8 T cells	Eotaxin	16	0.01	-0.61	1.80	–
Men	IFNα-pSTAT3-Monocytes	MIG	16	0.01	-0.68	1.61	–
Men	IFNα-pSTAT1-CD8 T cells	GP130	16	0.01	-0.66	1.22	–
Men	IL-10-pSTAT3-B cells	CRP	16	0.01	-0.71	1.04	–
Men	IL-10-pSTAT3-CD8 T cells	CRP	16	0.02	-0.68	1.05	–
Men	IFNγ-pSTAT1-B cells	I.309	16	0.02	-0.60	0.90	–
Men	IL-2-pSTAT5-CD4 T cells	BDNF	16	0.02	-0.65	0.95	–
Men	IFNα-pSTAT3-CD8 T cells	MIG	16	0.03	-0.44	1.01	–
Men	IFNα-pSTAT5-CD4 T cells	MIG	16	0.03	-0.53	0.92	–
Men	IL-10-pSTAT3-CD4 T cells	IP.10	16	0.03	-0.56	0.94	–
Men	IFNγ-pSTAT1-B cells	Eotaxin	16	0.03	-0.60	0.92	–
Men	IL-10-pSTAT3-B cells	Eotaxin	16	0.03	-0.52	0.95	–
Men	IL-10-pSTAT3-CD8 T cells	Eotaxin	16	0.04	-0.60	0.95	–
Men	IL-2-pSTAT5-CD4 T cells	MIG	16	0.04	-0.42	0.99	–
Men	IL-10-pSTAT3-CD4 T cells	Eotaxin	16	0.05	-0.53	1.14	–
Men	IFNα-pSTAT5-CD4 T cells	Eotaxin	16	0.05	-0.33	1.08	–
Men	IFNα-pSTAT3-CD8 T cells	I.TAC	16	0.05	-0.56	1.07	–
Men	IFNα-pSTAT3-CD8 T cells	I.309	16	0.06	-0.47	1.05	–
Men	IL-6-pSTAT3-Monocytes	I.TAC	16	0.06	-0.70	1.05	–
Men	IFNα-pSTAT3-Monocytes	sIL.6R	16	0.07	0.37	1.13	–
Men	IFNα-pSTAT3-B cells	Eotaxin	16	0.07	-0.32	1.15	–
Men	IFNα-pSTAT1-CD8 T cells	MCP.1	16	0.07	-0.53	1.13	–
Men	IL-2-pSTAT5-CD8 T cells	Eotaxin	16	0.07	-0.53	1.11	–
Men	IL-10-pSTAT3-CD8 T cells	IP.10	16	0.08	-0.39	1.09	–
Men	IFNα-pSTAT1-CD8 T cells	IL.10	14	0.08	0.39	1.09	–
Men	IFNα-pSTAT3-CD4 T cells	Eotaxin	16	0.09	-0.11	1.15	–
Men	IFNα-pSTAT5-CD4 T cells	BDNF	16	0.09	-0.45	1.19	–
Men	IL-10-pSTAT3-Monocytes	CRP	16	0.10	-0.36	1.19	–
Men	IL-2-pSTAT5-CD8 T cells	MIG	16	0.10	-0.18	1.19	–
Men	IFNα-pSTAT5-CD4 T cells	CRP	16	0.10	-0.48	1.16	–
Men	IFNγ-pSTAT1-B cells	CRP	16	0.10	-0.46	1.15	–
Men	IFNα-pSTAT3-B cells	MIG	16	0.11	-0.30	1.15	–
Men	IFNα-pSTAT3-B cells	I.309	16	0.11	-0.31	1.13	–
Men	IFNα-pSTAT3-Monocytes	BDNF	16	0.11	-0.24	1.12	–
Men	IL-6-pSTAT3-CD4 T cells	MIG	16	0.11	-0.46	1.13	–
Men	IFNγ-pSTAT1-B cells	IL.10	14	0.11	0.39	1.12	–
Men	IFNα-pSTAT5-CD4 T cells	I.309	16	0.11	-0.33	1.09	–
Men	IL-2-pSTAT5-CD4 T cells	Eotaxin	16	0.12	-0.33	1.08	–
Men	IL-6-pSTAT3-Monocytes	GP130	16	0.12	-0.53	1.07	–
Men	IL-10-pSTAT3-CD4 T cells	I.309	16	0.12	-0.44	1.05	–
Men	IFNα-pSTAT5-CD4 T cells	I.TAC	16	0.12	-0.38	1.03	–
Men	IFNα-pSTAT3-Monocytes	I.TAC	16	0.12	-0.44	1.02	–
Men	IL-2-pSTAT5-CD4 T cells	RANTES	16	0.13	-0.47	1.04	–
Men	IFNα-pSTAT5-CD4 T cells	GP130	16	0.13	-0.34	1.02	–

Table S8: *(continued)*

Sex	Stimulated condition	Marker	n	P value	rho	FDR	Selected
Men	IL-2-pSTAT5-CD4 T cells	I.309	16	0.13	-0.43	1.01	–
Men	IL-6-pSTAT1-CD4 T cells	IL.6	14	0.13	0.14	1.02	–
Men	IL-10-pSTAT3-CD4 T cells	MIG	16	0.13	-0.36	1.00	–
Men	IL-10-pSTAT3-CD8 T cells	I.TAC	16	0.14	-0.31	1.01	–
Men	IFNα-pSTAT3-CD8 T cells	BDNF	16	0.15	-0.24	1.09	–
Men	IFNα-pSTAT3-CD8 T cells	GP130	16	0.15	-0.45	1.07	–
Men	IFNα-pSTAT1-CD4 T cells	BDNF	16	0.15	-0.20	1.06	–
Men	IFNα-pSTAT3-Monocytes	Eotaxin	16	0.16	-0.17	1.06	–
Men	IL-2-pSTAT5-CD8 T cells	I.TAC	16	0.16	-0.21	1.05	–
Men	IFNα-pSTAT3-CD4 T cells	CRP	16	0.16	-0.22	1.04	–
Men	IFNα-pSTAT3-CD4 T cells	MIG	16	0.16	-0.43	1.05	–
Men	IFNα-pSTAT3-CD8 T cells	IL.10	14	0.17	0.45	1.05	–
Men	IL-2-pSTAT5-CD8 T cells	BDNF	16	0.17	-0.44	1.06	–
Men	IFNα-pSTAT3-CD8 T cells	sCD14	16	0.17	0.50	1.05	–
Men	IL-10-pSTAT3-Monocytes	IP.10	16	0.17	-0.35	1.03	–
Men	IL-6-pSTAT3-Monocytes	CD40L	16	0.17	-0.46	1.03	–
Men	IFNα-pSTAT3-Monocytes	P.sel	16	0.17	-0.52	1.01	–
Men	IFNγ-pSTAT1-B cells	RANTES	16	0.18	-0.31	1.01	–
Men	IL-10-pSTAT3-B cells	IP.10	16	0.18	-0.33	1.02	–
Men	IFNα-pSTAT1-CD4 T cells	GP130	16	0.19	-0.24	1.06	–
Men	IFNα-pSTAT3-CD8 T cells	CRP	16	0.19	-0.22	1.05	–
Men	IL-10-pSTAT3-B cells	sCD14	16	0.19	-0.28	1.04	–
Men	IFNγ-pSTAT1-Monocytes	Eotaxin	16	0.19	0.35	1.03	–
Men	IL-2-pSTAT5-CD8 T cells	sIL.6R	16	0.20	-0.32	1.02	–
Men	IFNα-pSTAT1-B cells	C.TACK	16	0.20	0.50	1.02	–
Men	IL-10-pSTAT3-CD8 T cells	MIG	16	0.20	-0.22	1.01	–
Men	IFNα-pSTAT1-B cells	BDNF	16	0.20	-0.22	1.02	–
Men	IFNα-pSTAT1-CD8 T cells	I.309	16	0.21	-0.39	1.04	–
Men	IL-10-pSTAT3-B cells	C.TACK	16	0.21	-0.29	1.04	–
Men	IL-2-pSTAT5-CD4 T cells	MCP.1	16	0.21	-0.44	1.02	–
Men	IFNγ-pSTAT1-Monocytes	BDNF	16	0.22	0.30	1.03	–
Men	IFNα-pSTAT1-CD8 T cells	BDNF	16	0.22	-0.32	1.02	–
Men	IL-10-pSTAT3-CD4 T cells	I.TAC	16	0.22	-0.28	1.01	–
Men	IFNα-pSTAT3-B cells	CRP	16	0.22	-0.34	1.02	–
Men	IL-2-pSTAT5-CD4 T cells	I.TAC	16	0.22	-0.26	1.01	–
Men	IL-10-pSTAT3-Monocytes	RANTES	16	0.23	0.29	1.00	–
Men	IFNα-pSTAT3-CD4 T cells	sIL.6R	16	0.23	0.23	1.00	–
Men	IL-10-pSTAT3-CD8 T cells	I.309	16	0.23	-0.32	1.01	–
Men	IFNγ-pSTAT1-B cells	sCD14	16	0.24	-0.34	1.02	–
Men	IL-6-pSTAT1-CD4 T cells	C5a	16	0.24	0.32	1.01	–
Men	IL-6-pSTAT3-CD4 T cells	P.sel	16	0.24	-0.37	1.02	–
Men	IFNα-pSTAT1-B cells	IP.10	16	0.25	0.25	1.03	–
Men	IFNα-pSTAT1-CD4 T cells	C5a	16	0.25	0.42	1.02	–
Men	IL-2-pSTAT5-CD4 T cells	GP130	16	0.25	-0.33	1.02	–
Men	IL-10-pSTAT3-B cells	RANTES	16	0.26	-0.27	1.04	–
Men	IFNα-pSTAT1-B cells	Eotaxin	16	0.26	-0.19	1.04	–

Table S8: *(continued)*

Sex	Stimulated condition	Marker	n	P value	rho	FDR	Selected
Men	IL-6-pSTAT3-CD4 T cells	C.TACK	16	0.26	0.33	1.03	–
Men	IL-6-pSTAT3-CD4 T cells	GP130	16	0.27	-0.22	1.03	–
Men	IFNγ-pSTAT1-Monocytes	GP130	16	0.27	-0.22	1.02	–
Men	IFNα-pSTAT1-CD4 T cells	Eotaxin	16	0.27	-0.09	1.02	–
Men	IFNγ-pSTAT1-B cells	C5a	16	0.28	-0.32	1.04	–
Men	IFNα-pSTAT1-CD8 T cells	Eotaxin	16	0.28	-0.31	1.03	–
Men	IFNα-pSTAT1-CD4 T cells	P.sel	16	0.28	-0.28	1.03	–
Men	IL-10-pSTAT3-B cells	CD40L	16	0.28	-0.15	1.03	–
Men	IFNα-pSTAT1-CD8 T cells	MIG	16	0.28	-0.31	1.02	–
Men	IL-6-pSTAT3-Monocytes	MIG	16	0.29	-0.36	1.05	–
Men	IFNα-pSTAT3-CD4 T cells	GP130	16	0.30	-0.23	1.05	–
Men	IFNα-pSTAT3-CD4 T cells	I.309	16	0.30	-0.08	1.04	–
Men	IL-10-pSTAT3-CD4 T cells	GP130	16	0.30	-0.31	1.03	–
Men	IFNα-pSTAT3-B cells	C.TACK	16	0.30	0.40	1.03	–
Men	IL-2-pSTAT5-CD8 T cells	I.309	16	0.30	-0.28	1.03	–
Men	IFNα-pSTAT3-B cells	GP130	16	0.31	-0.21	1.03	–
Men	IL-10-pSTAT3-B cells	I.309	16	0.31	-0.26	1.02	–
Men	IL-10-pSTAT3-CD8 T cells	IL.6	14	0.31	-0.31	1.01	–
Men	IFNα-pSTAT1-CD4 T cells	CRP	16	0.31	-0.22	1.01	–
Men	IFNα-pSTAT1-CD4 T cells	IL.6	14	0.31	0.19	1.02	–
Men	IFNα-pSTAT1-B cells	IL.6	14	0.32	0.07	1.02	–
Men	IFNγ-pSTAT1-B cells	GP130	16	0.32	-0.24	1.02	–
Men	IFNα-pSTAT1-CD8 T cells	I.TAC	16	0.32	-0.24	1.02	–
Men	IFNα-pSTAT1-CD4 T cells	MIG	16	0.32	-0.39	1.01	–
Men	IL-10-pSTAT3-CD4 T cells	sCD14	16	0.32	-0.33	1.00	–
Men	IFNα-pSTAT5-CD4 T cells	MCP.1	16	0.33	-0.31	1.01	–
Men	IL-6-pSTAT1-CD4 T cells	IP.10	16	0.33	0.23	1.00	–
Men	IL-6-pSTAT3-CD4 T cells	I.TAC	16	0.33	-0.23	1.00	–
Men	IFNα-pSTAT1-Monocytes	IP.10	16	0.33	0.25	1.00	–
Men	IFNα-pSTAT3-Monocytes	RANTES	16	0.34	-0.26	1.01	–
Men	IL-10-pSTAT3-B cells	I.TAC	16	0.34	-0.14	1.00	–
Men	IL-10-pSTAT3-Monocytes	P.sel	16	0.34	0.10	0.99	–
Men	IL-6-pSTAT3-CD4 T cells	CRP	16	0.35	-0.32	1.00	–
Men	IL-6-pSTAT3-CD4 T cells	sCD14	16	0.35	0.27	1.00	–
Men	IL-10-pSTAT3-Monocytes	IL.10	14	0.35	0.25	1.01	–
Men	IL-10-pSTAT3-Monocytes	Eotaxin	16	0.36	-0.28	1.03	–
Men	IL-10-pSTAT3-CD4 T cells	sIL.6R	16	0.36	-0.22	1.02	–
Men	IL-6-pSTAT3-CD4 T cells	I.309	16	0.36	-0.14	1.02	–
Men	IFNα-pSTAT1-B cells	GP130	16	0.37	-0.26	1.01	–
Men	IL-10-pSTAT3-Monocytes	CD40L	16	0.37	0.19	1.01	–
Men	IFNγ-pSTAT1-Monocytes	IL.6	14	0.37	0.23	1.00	–
Men	IL-2-pSTAT5-CD8 T cells	IP.10	16	0.37	-0.15	0.99	–
Men	IL-10-pSTAT3-CD8 T cells	C.TACK	16	0.38	-0.26	1.01	–
Men	IL-10-pSTAT3-Monocytes	sCD14	16	0.38	0.25	1.02	–
Men	IFNα-pSTAT3-CD4 T cells	BDNF	16	0.38	-0.05	1.01	–
Men	IFNα-pSTAT3-CD4 T cells	I.TAC	16	0.39	-0.18	1.02	–
Men	IFNα-pSTAT3-CD4 T cells	sCD14	16	0.39	0.22	1.02	–

Table S8: *(continued)*

Sex	Stimulated condition	Marker	n	P value	rho	FDR	Selected
Men	IFNα-pSTAT3-Monocytes	sCD14	16	0.40	0.26	1.04	–
Men	IFNα-pSTAT3-Monocytes	IL.6	14	0.40	-0.29	1.03	–
Men	IFNα-pSTAT1-CD8 T cells	IL.6	14	0.40	0.08	1.03	–
Men	IL-2-pSTAT5-CD4 T cells	sIL.6R	16	0.41	-0.32	1.04	–
Men	IL-10-pSTAT3-CD4 T cells	IL.6	14	0.41	-0.49	1.03	–
Men	IFNα-pSTAT3-Monocytes	GP130	16	0.41	-0.28	1.03	–
Men	IFNα-pSTAT1-B cells	I.309	16	0.41	-0.18	1.03	–
Men	IFNγ-pSTAT1-B cells	CD40L	16	0.41	-0.23	1.02	–
Men	IFNα-pSTAT1-Monocytes	C5a	16	0.42	-0.28	1.02	–
Men	IFNγ-pSTAT1-Monocytes	C.TACK	16	0.42	-0.12	1.03	–
Men	IL-10-pSTAT3-CD8 T cells	CD40L	16	0.42	-0.12	1.02	–
Men	IFNα-pSTAT1-CD4 T cells	I.TAC	16	0.43	-0.16	1.03	–
Men	IFNγ-pSTAT1-Monocytes	IL.10	14	0.43	0.07	1.02	–
Men	IL-2-pSTAT5-CD4 T cells	C5a	16	0.43	0.20	1.02	–
Men	IL-6-pSTAT3-Monocytes	MCP.1	16	0.43	0.21	1.02	–
Men	IL-2-pSTAT5-CD8 T cells	RANTES	16	0.44	-0.25	1.02	–
Men	IL-6-pSTAT3-Monocytes	IP.10	16	0.44	-0.27	1.02	–
Men	IL-6-pSTAT3-Monocytes	IL.10	14	0.45	0.18	1.03	–
Men	IL-6-pSTAT1-CD4 T cells	Eotaxin	16	0.45	0.15	1.03	–
Men	IL-6-pSTAT1-CD4 T cells	BDNF	16	0.45	-0.34	1.02	–
Men	IL-2-pSTAT5-CD8 T cells	CRP	16	0.46	-0.28	1.03	–
Men	IFNα-pSTAT1-CD8 T cells	P.sel	16	0.46	0.10	1.03	–
Men	IL-10-pSTAT3-Monocytes	GP130	16	0.46	-0.29	1.04	–
Men	IFNα-pSTAT1-Monocytes	GP130	16	0.46	-0.25	1.03	–
Men	IFNγ-pSTAT1-Monocytes	MCP.1	16	0.47	0.28	1.03	–
Men	IL-6-pSTAT3-CD4 T cells	Eotaxin	16	0.47	-0.06	1.03	–
Men	IFNα-pSTAT1-Monocytes	CRP	16	0.47	0.29	1.03	–
Men	IL-6-pSTAT3-CD4 T cells	CD40L	16	0.47	-0.06	1.02	–
Men	IFNγ-pSTAT1-Monocytes	sIL.6R	16	0.48	-0.30	1.03	–
Men	IFNα-pSTAT3-Monocytes	IP.10	16	0.48	-0.16	1.02	–
Men	IFNα-pSTAT1-B cells	IL.10	14	0.48	0.15	1.02	–
Men	IFNα-pSTAT3-Monocytes	I.309	16	0.48	-0.12	1.02	–
Men	IL-10-pSTAT3-B cells	sIL.6R	16	0.48	-0.16	1.01	–
Men	IFNγ-pSTAT1-B cells	BDNF	16	0.49	-0.14	1.02	–
Men	IFNα-pSTAT1-Monocytes	C.TACK	16	0.49	0.34	1.01	–
Men	IFNα-pSTAT1-CD4 T cells	sIL.6R	16	0.49	0.07	1.02	–
Men	IFNα-pSTAT1-CD8 T cells	sIL.6R	16	0.49	-0.31	1.01	–
Men	IL-10-pSTAT3-Monocytes	sIL.6R	16	0.50	-0.28	1.02	–
Men	IFNγ-pSTAT1-Monocytes	P.sel	16	0.50	0.11	1.01	–
Men	IFNα-pSTAT3-CD8 T cells	C5a	16	0.50	0.25	1.01	–
Men	IFNα-pSTAT1-B cells	RANTES	16	0.50	-0.24	1.01	–
Men	IL-6-pSTAT3-Monocytes	C5a	16	0.51	-0.12	1.01	–
Men	IL-6-pSTAT3-Monocytes	sIL.6R	16	0.51	-0.17	1.01	–
Men	IFNα-pSTAT5-CD4 T cells	C5a	16	0.51	0.31	1.01	–
Men	IL-10-pSTAT3-CD4 T cells	C.TACK	16	0.52	-0.23	1.02	–
Men	IFNα-pSTAT1-CD4 T cells	I.309	16	0.52	-0.05	1.02	–
Men	IFNα-pSTAT3-B cells	IL.10	14	0.53	0.22	1.02	–

Table S8: *(continued)*

Sex	Stimulated condition	Marker	n	P value	rho	FDR	Selected
Men	IFNγ-pSTAT1-B cells	MCP.1	16	0.53	-0.11	1.03	–
Men	IL-2-pSTAT5-CD4 T cells	CRP	16	0.53	-0.18	1.03	–
Men	IFNγ-pSTAT1-B cells	IL.6	14	0.54	0.03	1.03	–
Men	IL-10-pSTAT3-B cells	MIG	16	0.54	-0.09	1.02	–
Men	IFNα-pSTAT3-CD4 T cells	P.sel	16	0.54	-0.33	1.02	–
Men	IL-2-pSTAT5-CD8 T cells	MCP.1	16	0.54	-0.32	1.02	–
Men	IL-10-pSTAT3-CD4 T cells	BDNF	16	0.54	-0.28	1.01	–
Men	IFNα-pSTAT3-CD8 T cells	IP.10	16	0.54	-0.11	1.01	–
Men	IL-10-pSTAT3-B cells	C5a	16	0.55	-0.23	1.02	–
Men	IL-10-pSTAT3-Monocytes	I.309	16	0.55	-0.16	1.02	–
Men	IFNα-pSTAT3-Monocytes	CD40L	16	0.56	-0.10	1.02	–
Men	IFNα-pSTAT3-B cells	BDNF	16	0.56	0.08	1.02	–
Men	IFNγ-pSTAT1-Monocytes	MIG	16	0.56	0.14	1.03	–
Men	IFNα-pSTAT3-B cells	sIL.6R	16	0.57	0.16	1.02	–
Men	IFNα-pSTAT1-CD4 T cells	IP.10	16	0.57	0.20	1.02	–
Men	IFNα-pSTAT3-B cells	I.TAC	16	0.57	-0.09	1.03	–
Men	IL-2-pSTAT5-CD8 T cells	CD40L	16	0.57	-0.13	1.02	–
Men	IL-6-pSTAT3-CD4 T cells	sIL.6R	16	0.57	0.08	1.02	–
Men	IL-6-pSTAT3-CD4 T cells	C5a	16	0.58	0.21	1.02	–
Men	IFNα-pSTAT1-Monocytes	BDNF	16	0.58	0.10	1.02	–
Men	IL-2-pSTAT5-CD8 T cells	C5a	16	0.58	-0.01	1.02	–
Men	IFNα-pSTAT3-Monocytes	C.TACK	16	0.59	0.33	1.02	–
Men	IL-6-pSTAT1-CD4 T cells	sIL.6R	16	0.59	-0.17	1.02	–
Men	IFNα-pSTAT3-CD8 T cells	sIL.6R	16	0.59	0.19	1.01	–
Men	IFNα-pSTAT1-Monocytes	IL.10	14	0.59	-0.01	1.01	–
Men	IL-6-pSTAT1-CD4 T cells	GP130	16	0.59	-0.30	1.01	–
Men	IFNγ-pSTAT1-Monocytes	CRP	16	0.59	0.19	1.01	–
Men	IL-6-pSTAT1-CD4 T cells	MCP.1	16	0.59	-0.43	1.00	–
Men	IFNα-pSTAT1-Monocytes	IL.6	14	0.59	0.07	1.00	–
Men	IL-10-pSTAT3-CD4 T cells	C5a	16	0.60	-0.25	1.00	–
Men	IL-6-pSTAT3-CD4 T cells	IL.10	14	0.60	0.15	1.00	–
Men	IFNα-pSTAT1-CD4 T cells	sCD14	16	0.60	0.20	1.00	–
Men	IL-10-pSTAT3-Monocytes	C5a	16	0.60	-0.04	1.00	–
Men	IFNα-pSTAT1-Monocytes	P.sel	16	0.61	-0.17	1.00	–
Men	IL-10-pSTAT3-CD4 T cells	RANTES	16	0.61	-0.23	1.00	–
Men	IFNα-pSTAT1-CD4 T cells	RANTES	16	0.61	-0.08	1.00	–
Men	IL-10-pSTAT3-CD8 T cells	MCP.1	16	0.61	0.12	1.00	–
Men	IFNα-pSTAT3-Monocytes	MCP.1	16	0.62	-0.15	1.00	–
Men	IL-10-pSTAT3-CD4 T cells	MCP.1	16	0.62	-0.15	1.00	–
Men	IL-6-pSTAT1-CD4 T cells	P.sel	16	0.64	-0.18	1.02	–
Men	IL-10-pSTAT3-CD4 T cells	P.sel	16	0.64	0.16	1.03	–
Men	IFNα-pSTAT1-Monocytes	CD40L	16	0.64	-0.13	1.02	–
Men	IFNα-pSTAT1-B cells	MIG	16	0.64	-0.17	1.02	–
Men	IFNγ-pSTAT1-Monocytes	C5a	16	0.65	-0.21	1.02	–
Men	IFNα-pSTAT5-CD4 T cells	IL.6	14	0.65	0.01	1.01	–
Men	IL-6-pSTAT3-Monocytes	Eotaxin	16	0.65	-0.26	1.02	–

Table S8: *(continued)*

Sex	Stimulated condition	Marker	n	P value	rho	FDR	Selected
Men	IFNα-pSTAT1-CD4 T cells	CD40L	16	0.65	0.05	1.02	–
Men	IL-10-pSTAT3-CD4 T cells	CD40L	16	0.65	-0.15	1.01	–
Men	IFNα-pSTAT3-B cells	P.sel	16	0.66	-0.24	1.01	–
Men	IL-10-pSTAT3-Monocytes	BDNF	16	0.66	0.02	1.01	–
Men	IL-6-pSTAT3-Monocytes	I.309	16	0.67	-0.16	1.02	–
Men	IL-2-pSTAT5-CD4 T cells	sCD14	16	0.67	-0.21	1.02	–
Men	IL-6-pSTAT3-CD4 T cells	RANTES	16	0.68	0.13	1.02	–
Men	IFNα-pSTAT1-CD8 T cells	C.TACK	16	0.68	0.16	1.02	–
Men	IFNα-pSTAT3-B cells	C5a	16	0.68	-0.06	1.02	–
Men	IL-6-pSTAT1-CD4 T cells	sCD14	16	0.69	-0.01	1.03	–
Men	IL-6-pSTAT3-CD4 T cells	BDNF	16	0.69	0.05	1.03	–
Men	IL-6-pSTAT1-CD4 T cells	CRP	16	0.70	-0.02	1.04	–
Men	IL-2-pSTAT5-CD8 T cells	GP130	16	0.70	-0.09	1.04	–
Men	IFNγ-pSTAT1-Monocytes	CD40L	16	0.71	0.05	1.05	–
Men	IL-6-pSTAT3-Monocytes	IL.6	14	0.71	-0.25	1.04	–
Men	IFNα-pSTAT3-Monocytes	CRP	16	0.72	0.06	1.04	–
Men	IL-10-pSTAT3-Monocytes	MIG	16	0.72	-0.21	1.04	–
Men	IL-10-pSTAT3-B cells	IL.6	14	0.72	-0.26	1.04	–
Men	IFNα-pSTAT3-Monocytes	IL.10	14	0.72	-0.18	1.04	–
Men	IFNα-pSTAT1-CD8 T cells	CRP	16	0.73	-0.04	1.04	–
Men	IFNα-pSTAT5-CD4 T cells	sCD14	16	0.73	0.07	1.04	–
Men	IFNα-pSTAT3-CD8 T cells	MCP.1	16	0.73	0.04	1.04	–
Men	IFNα-pSTAT1-CD8 T cells	C5a	16	0.74	0.13	1.04	–
Men	IFNα-pSTAT1-Monocytes	I.309	16	0.74	-0.13	1.04	–
Men	IL-10-pSTAT3-Monocytes	I.TAC	16	0.74	-0.21	1.05	–
Men	IL-6-pSTAT3-Monocytes	CRP	16	0.74	-0.02	1.04	–
Men	IL-6-pSTAT3-Monocytes	sCD14	16	0.74	0.17	1.04	–
Men	IL-2-pSTAT5-CD4 T cells	IP.10	16	0.75	-0.11	1.04	–
Men	IFNα-pSTAT3-B cells	sCD14	16	0.75	0.18	1.04	–
Men	IL-2-pSTAT5-CD8 T cells	C.TACK	16	0.75	-0.08	1.04	–
Men	IFNα-pSTAT5-CD4 T cells	IP.10	16	0.75	-0.16	1.03	–
Men	IL-2-pSTAT5-CD8 T cells	IL.6	14	0.75	-0.12	1.03	–
Men	IFNα-pSTAT1-Monocytes	MCP.1	16	0.75	-0.11	1.03	–
Men	IFNα-pSTAT5-CD4 T cells	CD40L	16	0.76	-0.02	1.03	–
Men	IL-10-pSTAT3-B cells	GP130	16	0.76	0.03	1.03	–
Men	IFNα-pSTAT3-CD8 T cells	P.sel	16	0.76	-0.22	1.02	–
Men	IFNα-pSTAT1-Monocytes	sCD14	16	0.76	-0.02	1.03	–
Men	IFNα-pSTAT1-CD4 T cells	MCP.1	16	0.77	-0.16	1.03	–
Men	IFNα-pSTAT3-CD8 T cells	CD40L	16	0.77	-0.11	1.02	–
Men	IL-6-pSTAT1-CD4 T cells	I.TAC	16	0.77	-0.10	1.02	–
Men	IFNγ-pSTAT1-B cells	sIL.6R	16	0.77	-0.05	1.02	–
Men	IFNγ-pSTAT1-B cells	MIG	16	0.78	0.08	1.02	–
Men	IL-2-pSTAT5-CD8 T cells	P.sel	16	0.78	0.14	1.02	–
Men	IL-2-pSTAT5-CD4 T cells	IL.6	14	0.78	-0.12	1.03	–
Men	IL-10-pSTAT3-CD8 T cells	RANTES	16	0.79	-0.06	1.02	–
Men	IL-10-pSTAT3-B cells	IL.10	14	0.79	-0.07	1.03	–
Men	IFNγ-pSTAT1-B cells	P.sel	16	0.79	0.08	1.02	–

Table S8: *(continued)*

Sex	Stimulated condition	Marker	n	P value	rho	FDR	Selected
Men	IFNγ-pSTAT1-B cells	IP.10	16	0.79	0.19	1.02	–
Men	IFNγ-pSTAT1-Monocytes	I.TAC	16	0.79	0.07	1.02	–
Men	IFNα-pSTAT5-CD4 T cells	C.TACK	16	0.79	-0.24	1.01	–
Men	IFNα-pSTAT1-B cells	sIL.6R	16	0.79	0.01	1.01	–
Men	IL-6-pSTAT1-CD4 T cells	I.309	16	0.80	-0.09	1.02	–
Men	IL-10-pSTAT3-CD8 T cells	P.sel	16	0.80	-0.06	1.02	–
Men	IFNα-pSTAT3-CD4 T cells	IL.10	14	0.80	-0.03	1.02	–
Men	IL-10-pSTAT3-CD8 T cells	C5a	16	0.81	-0.13	1.02	–
Men	IFNα-pSTAT5-CD4 T cells	P.sel	16	0.81	0.02	1.02	–
Men	IL-2-pSTAT5-CD4 T cells	CD40L	16	0.82	-0.12	1.02	–
Men	IL-2-pSTAT5-CD8 T cells	sCD14	16	0.82	-0.14	1.02	–
Men	IL-10-pSTAT3-Monocytes	IL.6	14	0.82	-0.18	1.02	–
Men	IL-2-pSTAT5-CD4 T cells	C.TACK	16	0.82	0.01	1.01	–
Men	IL-10-pSTAT3-CD8 T cells	sIL.6R	16	0.82	0.06	1.01	–
Men	IL-10-pSTAT3-B cells	P.sel	16	0.83	-0.03	1.01	–
Men	IFNα-pSTAT3-B cells	CD40L	16	0.83	0.10	1.01	–
Men	IFNγ-pSTAT1-B cells	C.TACK	16	0.83	0.03	1.01	–
Men	IL-10-pSTAT3-CD8 T cells	sCD14	16	0.83	-0.02	1.01	–
Men	IFNα-pSTAT3-CD4 T cells	RANTES	16	0.83	-0.01	1.01	–
Men	IL-10-pSTAT3-B cells	MCP.1	16	0.83	0.06	1.01	–
Men	IFNα-pSTAT5-CD4 T cells	RANTES	16	0.83	-0.11	1.00	–
Men	IFNα-pSTAT1-CD8 T cells	IP.10	16	0.83	-0.05	1.00	–
Men	IL-6-pSTAT3-CD4 T cells	IL.6	14	0.84	-0.11	1.00	–
Men	IFNα-pSTAT1-B cells	CD40L	16	0.84	-0.07	1.00	–
Men	IFNα-pSTAT1-Monocytes	MIG	16	0.84	-0.21	1.00	–
Men	IFNα-pSTAT3-CD4 T cells	MCP.1	16	0.85	-0.05	1.00	–
Men	IFNα-pSTAT1-B cells	sCD14	16	0.85	0.15	1.00	–
Men	IFNα-pSTAT3-Monocytes	C5a	16	0.85	0.18	1.00	–
Men	IFNγ-pSTAT1-B cells	I.TAC	16	0.85	0.16	0.99	–
Men	IL-6-pSTAT1-CD4 T cells	C.TACK	16	0.85	0.24	0.99	–
Men	IL-10-pSTAT3-CD8 T cells	GP130	16	0.85	-0.06	0.99	–
Men	IFNα-pSTAT3-B cells	RANTES	16	0.85	0.05	0.99	–
Men	IFNα-pSTAT3-CD8 T cells	IL.6	14	0.85	0.03	0.99	–
Men	IL-2-pSTAT5-CD8 T cells	IL.10	14	0.86	-0.11	0.99	–
Men	IFNα-pSTAT1-B cells	CRP	16	0.86	-0.01	0.99	–
Men	IFNα-pSTAT1-B cells	P.sel	16	0.86	-0.20	0.99	–
Men	IFNα-pSTAT1-CD8 T cells	CD40L	16	0.86	-0.12	0.99	–
Men	IFNα-pSTAT3-CD8 T cells	RANTES	16	0.88	0.11	1.00	–
Men	IL-10-pSTAT3-CD8 T cells	BDNF	16	0.88	-0.11	1.00	–
Men	IL-2-pSTAT5-CD4 T cells	P.sel	16	0.88	0.12	1.00	–
Men	IL-10-pSTAT3-Monocytes	C.TACK	16	0.88	0.11	1.00	–
Men	IL-10-pSTAT3-B cells	BDNF	16	0.88	-0.11	0.99	–
Men	IL-2-pSTAT5-CD4 T cells	IL.10	14	0.89	-0.11	0.99	–
Men	IFNα-pSTAT5-CD4 T cells	IL.10	14	0.89	-0.01	0.99	–
Men	IFNα-pSTAT3-B cells	MCP.1	16	0.89	0.10	0.99	–
Men	IFNα-pSTAT1-CD8 T cells	sCD14	16	0.89	-0.09	0.99	–
Men	IFNα-pSTAT3-CD4 T cells	CD40L	16	0.89	0.10	0.99	–

Table S8: *(continued)*

Sex	Stimulated condition	Marker	n	P value	rho	FDR	Selected
Men	IFNα-pSTAT1-Monocytes	Eotaxin	16	0.89	0.04	0.98	–
Men	IFNγ-pSTAT1-Monocytes	sCD14	16	0.89	-0.13	0.98	–
Men	IL-6-pSTAT3-Monocytes	RANTES	16	0.90	-0.13	0.98	–
Men	IFNα-pSTAT1-Monocytes	sIL.6R	16	0.90	-0.04	0.99	–
Men	IFNα-pSTAT1-Monocytes	RANTES	16	0.90	-0.09	0.98	–
Men	IFNγ-pSTAT1-Monocytes	IP.10	16	0.90	0.09	0.98	–
Men	IL-10-pSTAT3-CD8 T cells	IL.10	14	0.90	0.06	0.98	–
Men	IFNα-pSTAT1-B cells	MCP.1	16	0.91	0.12	0.98	–
Men	IFNα-pSTAT5-CD4 T cells	sIL.6R	16	0.91	-0.24	0.98	–
Men	IFNα-pSTAT3-B cells	IL.6	14	0.92	-0.13	0.98	–
Men	IFNα-pSTAT3-CD4 T cells	IL.6	14	0.92	0.07	0.98	–
Men	IFNα-pSTAT3-CD4 T cells	IP.10	16	0.92	0.09	0.98	–
Men	IL-6-pSTAT3-Monocytes	C.TACK	16	0.92	0.01	0.98	–
Men	IL-10-pSTAT3-CD4 T cells	IL.10	14	0.93	0.08	0.99	–
Men	IL-6-pSTAT1-CD4 T cells	CD40L	16	0.93	-0.24	0.99	–
Men	IL-6-pSTAT1-CD4 T cells	RANTES	16	0.93	-0.25	0.99	–
Men	IFNα-pSTAT3-CD8 T cells	C.TACK	16	0.94	0.04	0.99	–
Men	IFNγ-pSTAT1-Monocytes	RANTES	16	0.94	0.02	0.99	–
Men	IL-6-pSTAT3-Monocytes	P.sel	16	0.94	-0.13	0.98	–
Men	IL-6-pSTAT3-CD4 T cells	IP.10	16	0.94	-0.04	0.98	–
Men	IFNα-pSTAT1-B cells	C5a	16	0.94	0.19	0.98	–
Men	IFNα-pSTAT1-Monocytes	I.TAC	16	0.94	-0.03	0.98	–
Men	IFNα-pSTAT3-CD4 T cells	C5a	16	0.95	0.25	0.98	–
Men	IL-6-pSTAT1-CD4 T cells	IL.10	14	0.96	0.07	0.99	–
Men	IL-6-pSTAT1-CD4 T cells	MIG	16	0.97	-0.40	1.00	–
Men	IL-6-pSTAT3-Monocytes	BDNF	16	0.97	-0.18	1.00	–
Men	IFNγ-pSTAT1-Monocytes	I.309	16	0.97	-0.01	1.00	–
Men	IL-10-pSTAT3-Monocytes	MCP.1	16	0.97	-0.05	0.99	–
Men	IFNα-pSTAT1-CD8 T cells	RANTES	16	0.98	-0.09	0.99	–
Men	IFNα-pSTAT1-CD4 T cells	C.TACK	16	0.99	0.06	1.00	–
Men	IFNα-pSTAT1-CD4 T cells	IL.10	14	0.99	-0.03	1.00	–
Men	IFNα-pSTAT3-CD4 T cells	C.TACK	16	0.99	0.11	1.00	–
Men	IL-6-pSTAT3-CD4 T cells	MCP.1	16	0.99	-0.05	1.00	–
Men	IFNα-pSTAT1-B cells	I.TAC	16	1.00	-0.12	1.00	–
Men	IFNα-pSTAT3-B cells	IP.10	16	1.00	0.00	1.00	–
Women	IL-6-pSTAT3-CD4 T cells	GP130	17	0.00	0.72	0.66	–
Women	IL-6-pSTAT1-CD4 T cells	MCP.1	18	0.01	0.66	1.04	–
Women	IL-6-pSTAT1-CD4 T cells	GP130	17	0.01	0.50	1.31	–
Women	IL-2-pSTAT5-CD4 T cells	CRP	18	0.02	-0.45	1.41	–
Women	IFNα-pSTAT1-Monocytes	CRP	18	0.02	-0.63	1.42	–
Women	IFNα-pSTAT1-CD4 T cells	Eotaxin	18	0.02	0.51	1.34	–
Women	IL-6-pSTAT1-CD4 T cells	Eotaxin	18	0.02	0.52	1.23	–
Women	IL-10-pSTAT3-Monocytes	sCD14	18	0.03	-0.57	1.38	–
Women	IFNα-pSTAT3-CD8 T cells	sIL.6R	18	0.03	-0.67	1.27	–
Women	IL-6-pSTAT3-CD4 T cells	MCP.1	18	0.03	0.54	1.25	–
Women	IL-10-pSTAT3-CD8 T cells	Eotaxin	18	0.04	0.53	1.26	–

Table S8: *(continued)*

Sex	Stimulated condition	Marker	n	P value	rho	FDR	Selected
Women	IL-10-pSTAT3-CD8 T cells	C.TACK	18	0.04	-0.47	1.21	–
Women	IL-6-pSTAT3-Monocytes	MCP.1	18	0.04	0.51	1.12	–
Women	IFNα-pSTAT3-CD4 T cells	Eotaxin	18	0.04	0.48	1.10	–
Women	IL-10-pSTAT3-Monocytes	CRP	18	0.04	-0.30	1.05	–
Women	IFNγ-pSTAT1-B cells	BDNF	18	0.04	-0.54	0.99	–
Women	IL-10-pSTAT3-CD4 T cells	C5a	18	0.05	-0.46	1.03	–
Women	IFNα-pSTAT1-B cells	sCD14	18	0.05	0.49	1.00	–
Women	IFNα-pSTAT5-CD4 T cells	CRP	18	0.05	-0.35	0.95	–
Women	IL-2-pSTAT5-CD4 T cells	IL.10	17	0.05	0.44	0.91	–
Women	IFNα-pSTAT3-CD8 T cells	Eotaxin	18	0.05	0.49	0.91	–
Women	IL-6-pSTAT1-CD4 T cells	I.TAC	18	0.06	0.44	1.03	–
Women	IFNα-pSTAT3-B cells	I.TAC	18	0.06	0.33	1.02	–
Women	IL-6-pSTAT1-CD4 T cells	MIG	18	0.07	0.35	1.11	–
Women	IL-10-pSTAT3-Monocytes	CD40L	18	0.08	0.37	1.09	–
Women	IL-10-pSTAT3-Monocytes	C.TACK	18	0.08	-0.41	1.07	–
Women	IL-2-pSTAT5-CD4 T cells	CD40L	18	0.09	0.39	1.16	–
Women	IFNα-pSTAT1-B cells	Eotaxin	18	0.09	0.51	1.15	–
Women	IFNα-pSTAT3-B cells	Eotaxin	18	0.09	0.34	1.15	–
Women	IFNα-pSTAT3-CD8 T cells	CRP	18	0.10	-0.62	1.16	–
Women	IFNγ-pSTAT1-Monocytes	sIL.6R	18	0.10	-0.50	1.14	–
Women	IL-10-pSTAT3-B cells	C.TACK	18	0.10	-0.34	1.10	–
Women	IL-10-pSTAT3-CD4 T cells	I.TAC	18	0.10	0.43	1.09	–
Women	IFNα-pSTAT3-CD4 T cells	MCP.1	18	0.10	0.32	1.08	–
Women	IL-6-pSTAT3-CD4 T cells	Eotaxin	18	0.10	0.37	1.07	–
Women	IL-6-pSTAT3-CD4 T cells	I.TAC	18	0.11	0.42	1.06	–
Women	IFNα-pSTAT3-Monocytes	I.TAC	18	0.11	0.36	1.05	–
Women	IFNα-pSTAT1-CD4 T cells	MCP.1	18	0.11	0.40	1.04	–
Women	IFNγ-pSTAT1-Monocytes	IL.6	17	0.11	0.28	1.03	–
Women	IL-6-pSTAT3-CD4 T cells	sCD14	18	0.11	0.36	1.02	–
Women	IFNα-pSTAT3-CD8 T cells	I.TAC	18	0.11	0.41	1.00	–
Women	IFNα-pSTAT1-CD8 T cells	I.TAC	18	0.11	0.37	0.98	–
Women	IFNα-pSTAT1-B cells	BDNF	18	0.11	-0.29	0.96	–
Women	IFNγ-pSTAT1-B cells	sIL.6R	18	0.12	-0.38	0.94	–
Women	IL-2-pSTAT5-CD8 T cells	CRP	18	0.12	-0.44	0.93	–
Women	IFNγ-pSTAT1-B cells	RANTES	18	0.12	-0.40	0.94	–
Women	IFNα-pSTAT1-CD4 T cells	RANTES	18	0.12	0.40	0.93	–
Women	IL-6-pSTAT3-CD4 T cells	IP.10	18	0.13	0.37	0.95	–
Women	IFNα-pSTAT1-Monocytes	C.TACK	18	0.13	-0.26	0.94	–
Women	IL-2-pSTAT5-CD4 T cells	MIG	18	0.13	0.28	0.92	–
Women	IL-10-pSTAT3-CD8 T cells	sCD14	18	0.13	-0.34	0.91	–
Women	IFNγ-pSTAT1-Monocytes	sCD14	18	0.13	-0.43	0.89	–
Women	IFNα-pSTAT3-B cells	CRP	18	0.13	-0.34	0.87	–
Women	IL-6-pSTAT3-CD4 T cells	I.309	18	0.13	0.34	0.87	–
Women	IFNγ-pSTAT1-Monocytes	CRP	18	0.13	-0.33	0.85	–
Women	IFNγ-pSTAT1-B cells	GP130	17	0.13	0.47	0.86	–
Women	IL-10-pSTAT3-CD4 T cells	sIL.6R	18	0.14	-0.42	0.86	–
Women	IFNα-pSTAT1-B cells	IL.6	17	0.14	-0.25	0.88	–

Table S8: *(continued)*

Sex	Stimulated condition	Marker	n	P value	rho	FDR	Selected
Women	IFNγ-pSTAT1-B cells	IP.10	18	0.14	0.47	0.88	–
Women	IFNα-pSTAT1-CD4 T cells	sCD14	18	0.15	0.41	0.89	–
Women	IFNα-pSTAT3-Monocytes	IL.6	17	0.15	0.32	0.87	–
Women	IL-6-pSTAT1-CD4 T cells	CRP	18	0.15	-0.47	0.88	–
Women	IFNα-pSTAT1-B cells	GP130	17	0.15	0.45	0.88	–
Women	IFNγ-pSTAT1-Monocytes	IL.10	17	0.16	0.33	0.90	–
Women	IL-10-pSTAT3-CD4 T cells	CD40L	18	0.16	0.40	0.89	–
Women	IL-6-pSTAT3-CD4 T cells	C5a	18	0.16	-0.30	0.88	–
Women	IL-10-pSTAT3-Monocytes	sIL.6R	18	0.16	-0.45	0.88	–
Women	IFNα-pSTAT5-CD4 T cells	MCP.1	18	0.17	0.33	0.89	–
Women	IL-10-pSTAT3-B cells	CRP	18	0.17	-0.26	0.88	–
Women	IFNα-pSTAT1-CD4 T cells	MIG	18	0.17	0.29	0.88	–
Women	IFNα-pSTAT3-Monocytes	CRP	18	0.17	-0.24	0.87	–
Women	IFNα-pSTAT5-CD4 T cells	IP.10	18	0.17	-0.20	0.86	–
Women	IL-6-pSTAT1-CD4 T cells	I.309	18	0.17	0.16	0.85	–
Women	IL-2-pSTAT5-CD8 T cells	CD40L	18	0.18	0.28	0.87	–
Women	IFNα-pSTAT1-Monocytes	MCP.1	18	0.19	0.35	0.90	–
Women	IFNα-pSTAT3-CD8 T cells	IL.6	17	0.19	0.34	0.89	–
Women	IL-10-pSTAT3-CD8 T cells	sIL.6R	18	0.19	-0.32	0.88	–
Women	IL-10-pSTAT3-CD8 T cells	BDNF	18	0.19	0.27	0.88	–
Women	IFNα-pSTAT1-B cells	CD40L	18	0.20	-0.24	0.91	–
Women	IL-2-pSTAT5-CD4 T cells	Eotaxin	18	0.20	-0.34	0.90	–
Women	IL-10-pSTAT3-CD8 T cells	C5a	18	0.20	-0.29	0.90	–
Women	IL-6-pSTAT3-CD4 T cells	MIG	18	0.20	0.23	0.89	–
Women	IL-6-pSTAT3-Monocytes	CRP	18	0.20	-0.31	0.88	–
Women	IFNγ-pSTAT1-B cells	CRP	18	0.21	-0.47	0.89	–
Women	IL-6-pSTAT1-CD4 T cells	IL.6	17	0.21	0.31	0.90	–
Women	IFNα-pSTAT3-CD4 T cells	I.TAC	18	0.21	0.16	0.89	–
Women	IFNα-pSTAT1-CD4 T cells	C5a	18	0.21	-0.35	0.88	–
Women	IFNα-pSTAT1-CD8 T cells	Eotaxin	18	0.21	0.29	0.88	–
Women	IFNα-pSTAT1-CD8 T cells	sIL.6R	18	0.22	-0.32	0.89	–
Women	IFNα-pSTAT1-CD8 T cells	GP130	17	0.22	0.32	0.88	–
Women	IL-6-pSTAT1-CD4 T cells	C5a	18	0.22	-0.37	0.87	–
Women	IL-2-pSTAT5-CD4 T cells	I.309	18	0.22	0.40	0.87	–
Women	IFNα-pSTAT1-Monocytes	IL.10	17	0.22	0.46	0.87	–
Women	IL-2-pSTAT5-CD8 T cells	IL.10	17	0.23	0.19	0.86	–
Women	IL-10-pSTAT3-Monocytes	IL.6	17	0.23	0.28	0.86	–
Women	IFNα-pSTAT3-B cells	I.309	18	0.23	0.22	0.85	–
Women	IL-2-pSTAT5-CD8 T cells	sCD14	18	0.24	-0.08	0.87	–
Women	IFNα-pSTAT1-Monocytes	I.TAC	18	0.24	0.28	0.88	–
Women	IL-2-pSTAT5-CD4 T cells	sCD14	18	0.24	-0.24	0.87	–
Women	IFNα-pSTAT3-Monocytes	MCP.1	18	0.24	0.22	0.88	–
Women	IL-2-pSTAT5-CD4 T cells	IL.6	17	0.24	0.26	0.87	–
Women	IFNα-pSTAT1-CD8 T cells	BDNF	18	0.25	-0.25	0.87	–
Women	IL-10-pSTAT3-CD8 T cells	CRP	18	0.25	-0.37	0.87	–
Women	IFNα-pSTAT1-Monocytes	IL.6	17	0.25	0.23	0.86	–
Women	IL-6-pSTAT3-Monocytes	RANTES	18	0.26	-0.19	0.88	–

Table S8: *(continued)*

Sex	Stimulated condition	Marker	n	P value	rho	FDR	Selected
Women	IFNα-pSTAT1-CD8 T cells	IP.10	18	0.26	0.27	0.89	–
Women	IL-6-pSTAT1-CD4 T cells	IP.10	18	0.26	0.06	0.88	–
Women	IL-10-pSTAT3-CD8 T cells	I.TAC	18	0.27	0.34	0.89	–
Women	IL-10-pSTAT3-B cells	CD40L	18	0.27	0.21	0.88	–
Women	IFNα-pSTAT1-CD8 T cells	C5a	18	0.27	-0.26	0.88	–
Women	IFNα-pSTAT1-CD4 T cells	I.TAC	18	0.27	0.27	0.88	–
Women	IL-10-pSTAT3-Monocytes	IL.10	17	0.27	0.28	0.88	–
Women	IFNα-pSTAT1-Monocytes	sCD14	18	0.28	-0.30	0.89	–
Women	IFNγ-pSTAT1-Monocytes	MIG	18	0.28	0.20	0.89	–
Women	IL-2-pSTAT5-CD8 T cells	GP130	17	0.28	0.22	0.89	–
Women	IL-6-pSTAT1-CD4 T cells	sCD14	18	0.28	0.35	0.88	–
Women	IL-6-pSTAT1-CD4 T cells	CD40L	18	0.29	0.26	0.88	–
Women	IL-2-pSTAT5-CD8 T cells	Eotaxin	18	0.29	-0.17	0.88	–
Women	IL-6-pSTAT1-CD4 T cells	RANTES	18	0.29	0.23	0.87	–
Women	IFNα-pSTAT1-Monocytes	GP130	17	0.30	-0.12	0.89	–
Women	IFNα-pSTAT3-CD4 T cells	RANTES	18	0.30	0.25	0.89	–
Women	IFNα-pSTAT3-CD8 T cells	MCP.1	18	0.30	0.33	0.89	–
Women	IL-6-pSTAT1-CD4 T cells	IL.10	17	0.30	0.26	0.89	–
Women	IFNα-pSTAT3-CD4 T cells	C5a	18	0.30	-0.20	0.88	–
Women	IFNα-pSTAT3-CD4 T cells	CRP	18	0.30	-0.24	0.88	–
Women	IFNα-pSTAT5-CD4 T cells	C5a	18	0.31	0.38	0.88	–
Women	IL-10-pSTAT3-CD8 T cells	CD40L	18	0.32	0.17	0.90	–
Women	IFNα-pSTAT3-B cells	MIG	18	0.32	-0.01	0.89	–
Women	IFNα-pSTAT1-CD4 T cells	CRP	18	0.32	-0.35	0.89	–
Women	IL-2-pSTAT5-CD4 T cells	MCP.1	18	0.32	0.13	0.89	–
Women	IFNγ-pSTAT1-Monocytes	C.TACK	18	0.32	-0.16	0.89	–
Women	IL-10-pSTAT3-Monocytes	GP130	17	0.33	-0.33	0.89	–
Women	IL-2-pSTAT5-CD4 T cells	I.TAC	18	0.33	0.28	0.89	–
Women	IFNα-pSTAT1-Monocytes	Eotaxin	18	0.33	0.22	0.89	–
Women	IFNα-pSTAT1-B cells	C5a	18	0.33	-0.03	0.88	–
Women	IFNα-pSTAT3-Monocytes	C5a	18	0.34	-0.12	0.89	–
Women	IL-2-pSTAT5-CD8 T cells	BDNF	18	0.34	-0.32	0.89	–
Women	IL-10-pSTAT3-CD4 T cells	C.TACK	18	0.34	-0.15	0.88	–
Women	IL-10-pSTAT3-Monocytes	IP.10	18	0.34	-0.07	0.88	–
Women	IL-6-pSTAT3-CD4 T cells	P.sel	18	0.34	0.21	0.88	–
Women	IFNγ-pSTAT1-B cells	MCP.1	18	0.34	0.37	0.88	–
Women	IFNα-pSTAT3-Monocytes	IL.10	17	0.34	0.31	0.87	–
Women	IFNα-pSTAT3-CD8 T cells	RANTES	18	0.34	0.15	0.87	–
Women	IFNα-pSTAT3-CD4 T cells	IP.10	18	0.35	-0.32	0.87	–
Women	IFNα-pSTAT1-B cells	IL.10	17	0.35	-0.22	0.86	–
Women	IL-2-pSTAT5-CD4 T cells	P.sel	18	0.35	0.18	0.87	–
Women	IL-10-pSTAT3-CD8 T cells	GP130	17	0.35	-0.20	0.86	–
Women	IL-10-pSTAT3-CD8 T cells	RANTES	18	0.35	0.19	0.86	–
Women	IFNα-pSTAT1-B cells	C.TACK	18	0.35	0.15	0.85	–
Women	IL-10-pSTAT3-CD4 T cells	Eotaxin	18	0.36	0.21	0.85	–
Women	IL-6-pSTAT3-CD4 T cells	CD40L	18	0.36	0.28	0.85	–

Table S8: *(continued)*

Sex	Stimulated condition	Marker	n	P value	rho	FDR	Selected
Women	IFNα-pSTAT1-B cells	IP.10	18	0.37	0.19	0.87	–
Women	IL-6-pSTAT3-Monocytes	C.TACK	18	0.37	-0.17	0.87	–
Women	IL-10-pSTAT3-B cells	I.TAC	18	0.37	0.23	0.87	–
Women	IFNα-pSTAT1-B cells	RANTES	18	0.38	-0.16	0.87	–
Women	IL-2-pSTAT5-CD4 T cells	C.TACK	18	0.38	-0.28	0.88	–
Women	IFNα-pSTAT1-B cells	MCP.1	18	0.38	0.39	0.87	–
Women	IFNα-pSTAT1-Monocytes	MIG	18	0.39	0.29	0.88	–
Women	IFNα-pSTAT3-CD8 T cells	MIG	18	0.39	0.21	0.88	–
Women	IFNα-pSTAT3-CD8 T cells	IL.10	17	0.39	0.29	0.88	–
Women	IL-2-pSTAT5-CD8 T cells	P.sel	18	0.39	0.28	0.87	–
Women	IL-10-pSTAT3-B cells	P.sel	18	0.39	0.29	0.87	–
Women	IL-10-pSTAT3-CD4 T cells	CRP	18	0.40	-0.27	0.87	–
Women	IFNα-pSTAT3-B cells	MCP.1	18	0.40	0.06	0.87	–
Women	IL-6-pSTAT3-CD4 T cells	RANTES	18	0.40	0.05	0.87	–
Women	IL-10-pSTAT3-B cells	GP130	17	0.40	-0.15	0.87	–
Women	IFNα-pSTAT3-Monocytes	GP130	17	0.40	-0.06	0.87	–
Women	IFNα-pSTAT3-Monocytes	IP.10	18	0.41	-0.19	0.88	–
Women	IL-2-pSTAT5-CD8 T cells	I.309	18	0.41	0.09	0.88	–
Women	IL-6-pSTAT1-CD4 T cells	P.sel	18	0.41	0.21	0.87	–
Women	IFNα-pSTAT3-CD8 T cells	C5a	18	0.41	-0.24	0.87	–
Women	IFNα-pSTAT1-CD4 T cells	P.sel	18	0.41	0.25	0.87	–
Women	IFNα-pSTAT1-CD4 T cells	BDNF	18	0.42	0.22	0.86	–
Women	IL-2-pSTAT5-CD8 T cells	sIL.6R	18	0.42	-0.32	0.88	–
Women	IL-6-pSTAT3-Monocytes	C5a	18	0.42	-0.24	0.87	–
Women	IFNα-pSTAT3-Monocytes	Eotaxin	18	0.43	0.18	0.87	–
Women	IL-6-pSTAT1-CD4 T cells	BDNF	18	0.44	0.09	0.89	–
Women	IFNα-pSTAT3-Monocytes	P.sel	18	0.44	-0.12	0.89	–
Women	IFNα-pSTAT3-Monocytes	sCD14	18	0.44	-0.18	0.89	–
Women	IL-10-pSTAT3-B cells	RANTES	18	0.44	0.12	0.89	–
Women	IFNα-pSTAT3-B cells	GP130	17	0.45	0.17	0.89	–
Women	IFNγ-pSTAT1-B cells	Eotaxin	18	0.45	0.23	0.89	–
Women	IFNα-pSTAT5-CD4 T cells	BDNF	18	0.45	-0.28	0.88	–
Women	IFNα-pSTAT1-CD8 T cells	MCP.1	18	0.45	0.12	0.88	–
Women	IL-6-pSTAT3-CD4 T cells	IL.6	17	0.45	0.21	0.88	–
Women	IL-6-pSTAT3-CD4 T cells	BDNF	18	0.45	0.06	0.87	–
Women	IFNα-pSTAT3-Monocytes	RANTES	18	0.45	0.19	0.87	–
Women	IL-6-pSTAT3-Monocytes	P.sel	18	0.45	-0.24	0.87	–
Women	IFNα-pSTAT1-CD4 T cells	GP130	17	0.45	0.17	0.86	–
Women	IFNα-pSTAT1-Monocytes	sIL.6R	18	0.46	-0.29	0.86	–
Women	IFNα-pSTAT5-CD4 T cells	IL.10	17	0.46	0.00	0.86	–
Women	IL-10-pSTAT3-CD4 T cells	I.309	18	0.46	0.35	0.87	–
Women	IL-10-pSTAT3-Monocytes	I.TAC	18	0.46	0.15	0.86	–
Women	IL-2-pSTAT5-CD8 T cells	RANTES	18	0.46	-0.17	0.86	–
Women	IFNα-pSTAT5-CD4 T cells	RANTES	18	0.48	-0.25	0.88	–
Women	IFNα-pSTAT3-B cells	RANTES	18	0.48	0.09	0.88	–
Women	IFNγ-pSTAT1-Monocytes	MCP.1	18	0.48	0.15	0.88	–
Women	IL-6-pSTAT3-CD4 T cells	C.TACK	18	0.48	0.25	0.87	–

Table S8: *(continued)*

Sex	Stimulated condition	Marker	n	P value	rho	FDR	Selected
Women	IL-10-pSTAT3-CD4 T cells	RANTES	18	0.48	0.14	0.87	–
Women	IL-10-pSTAT3-CD4 T cells	sCD14	18	0.49	-0.12	0.88	–
Women	IFNα-pSTAT1-CD4 T cells	C.TACK	18	0.49	-0.29	0.87	–
Women	IFNα-pSTAT3-CD8 T cells	P.sel	18	0.49	0.11	0.88	–
Women	IFNγ-pSTAT1-Monocytes	I.TAC	18	0.50	0.00	0.89	–
Women	IFNγ-pSTAT1-Monocytes	I.309	18	0.50	0.05	0.89	–
Women	IL-2-pSTAT5-CD4 T cells	GP130	17	0.51	0.04	0.89	–
Women	IFNα-pSTAT1-CD8 T cells	CD40L	18	0.51	-0.15	0.89	–
Women	IL-6-pSTAT3-Monocytes	IL.10	17	0.51	0.10	0.89	–
Women	IL-10-pSTAT3-B cells	MIG	18	0.51	0.16	0.89	–
Women	IFNγ-pSTAT1-Monocytes	Eotaxin	18	0.52	0.16	0.90	–
Women	IL-10-pSTAT3-Monocytes	C5a	18	0.52	-0.18	0.90	–
Women	IFNα-pSTAT3-CD8 T cells	GP130	17	0.52	0.20	0.89	–
Women	IFNα-pSTAT5-CD4 T cells	sCD14	18	0.52	-0.14	0.89	–
Women	IL-6-pSTAT3-Monocytes	BDNF	18	0.53	-0.17	0.89	–
Women	IFNα-pSTAT3-CD4 T cells	I.309	18	0.53	0.06	0.89	–
Women	IL-10-pSTAT3-B cells	MCP.1	18	0.54	-0.33	0.90	–
Women	IFNα-pSTAT3-CD4 T cells	sCD14	18	0.54	0.19	0.89	–
Women	IL-2-pSTAT5-CD8 T cells	MIG	18	0.54	0.09	0.89	–
Women	IL-10-pSTAT3-B cells	IP.10	18	0.54	0.03	0.90	–
Women	IL-6-pSTAT3-CD4 T cells	CRP	18	0.54	-0.26	0.90	–
Women	IFNα-pSTAT1-CD8 T cells	C.TACK	18	0.54	0.13	0.89	–
Women	IL-2-pSTAT5-CD8 T cells	IP.10	18	0.55	0.17	0.89	–
Women	IFNα-pSTAT3-CD4 T cells	IL.6	17	0.55	0.12	0.89	–
Women	IL-10-pSTAT3-CD8 T cells	IL.6	17	0.55	0.21	0.89	–
Women	IL-10-pSTAT3-Monocytes	BDNF	18	0.55	0.03	0.89	–
Women	IFNα-pSTAT1-CD4 T cells	IL.6	17	0.56	0.22	0.89	–
Women	IL-10-pSTAT3-Monocytes	P.sel	18	0.56	0.05	0.89	–
Women	IL-2-pSTAT5-CD8 T cells	IL.6	17	0.57	0.06	0.90	–
Women	IL-10-pSTAT3-Monocytes	RANTES	18	0.57	0.05	0.90	–
Women	IL-2-pSTAT5-CD8 T cells	I.TAC	18	0.58	0.14	0.91	–
Women	IFNα-pSTAT3-CD4 T cells	BDNF	18	0.58	0.07	0.91	–
Women	IFNα-pSTAT5-CD4 T cells	C.TACK	18	0.58	-0.05	0.91	–
Women	IFNγ-pSTAT1-B cells	I.TAC	18	0.59	0.30	0.91	–
Women	IL-6-pSTAT3-CD4 T cells	IL.10	17	0.59	0.20	0.91	–
Women	IFNα-pSTAT1-Monocytes	IP.10	18	0.59	0.02	0.91	–
Women	IFNα-pSTAT1-CD8 T cells	MIG	18	0.60	-0.04	0.92	–
Women	IL-10-pSTAT3-B cells	sCD14	18	0.60	-0.10	0.91	–
Women	IFNα-pSTAT1-CD8 T cells	CRP	18	0.61	-0.20	0.92	–
Women	IFNγ-pSTAT1-B cells	IL.6	17	0.61	-0.15	0.92	–
Women	IFNα-pSTAT1-Monocytes	CD40L	18	0.61	-0.23	0.92	–
Women	IFNα-pSTAT5-CD4 T cells	GP130	17	0.62	-0.08	0.93	–
Women	IFNα-pSTAT1-CD8 T cells	P.sel	18	0.62	-0.14	0.92	–
Women	IFNα-pSTAT3-Monocytes	BDNF	18	0.62	0.09	0.92	–
Women	IFNα-pSTAT3-CD8 T cells	IP.10	18	0.62	-0.04	0.92	–
Women	IFNα-pSTAT3-CD8 T cells	I.309	18	0.64	0.00	0.94	–
Women	IFNγ-pSTAT1-B cells	MIG	18	0.64	0.07	0.94	–

Table S8: *(continued)*

Sex	Stimulated condition	Marker	n	P value	rho	FDR	Selected
Women	IL-6-pSTAT3-CD4 T cells	sIL.6R	18	0.65	-0.03	0.94	–
Women	IFNα-pSTAT1-B cells	CRP	18	0.65	-0.24	0.94	–
Women	IFNα-pSTAT1-B cells	MIG	18	0.65	0.01	0.94	–
Women	IL-2-pSTAT5-CD8 T cells	C5a	18	0.65	0.12	0.94	–
Women	IFNα-pSTAT3-B cells	sCD14	18	0.65	0.04	0.94	–
Women	IL-6-pSTAT3-Monocytes	I.309	18	0.66	0.00	0.94	–
Women	IL-10-pSTAT3-Monocytes	MCP.1	18	0.66	-0.18	0.94	–
Women	IFNα-pSTAT1-Monocytes	P.sel	18	0.66	-0.05	0.94	–
Women	IFNγ-pSTAT1-Monocytes	GP130	17	0.66	-0.14	0.94	–
Women	IL-6-pSTAT3-Monocytes	sIL.6R	18	0.67	-0.23	0.94	–
Women	IFNα-pSTAT5-CD4 T cells	IL.6	17	0.67	-0.03	0.95	–
Women	IFNα-pSTAT3-CD4 T cells	C.TACK	18	0.68	-0.11	0.95	–
Women	IL-10-pSTAT3-CD4 T cells	IL.6	17	0.69	0.20	0.96	–
Women	IL-10-pSTAT3-CD4 T cells	BDNF	18	0.70	0.05	0.97	–
Women	IFNα-pSTAT5-CD4 T cells	CD40L	18	0.70	0.12	0.97	–
Women	IFNα-pSTAT1-CD8 T cells	I.309	18	0.70	0.05	0.97	–
Women	IFNα-pSTAT1-CD4 T cells	sIL.6R	18	0.71	-0.03	0.97	–
Women	IFNα-pSTAT1-Monocytes	C5a	18	0.71	0.08	0.97	–
Women	IL-2-pSTAT5-CD4 T cells	sIL.6R	18	0.71	-0.11	0.97	–
Women	IFNγ-pSTAT1-Monocytes	CD40L	18	0.72	-0.15	0.97	–
Women	IL-2-pSTAT5-CD4 T cells	BDNF	18	0.72	-0.19	0.97	–
Women	IFNα-pSTAT3-B cells	C.TACK	18	0.72	0.08	0.97	–
Women	IFNα-pSTAT1-B cells	I.309	18	0.72	-0.29	0.97	–
Women	IFNα-pSTAT3-CD4 T cells	MIG	18	0.72	0.00	0.96	–
Women	IFNα-pSTAT3-Monocytes	I.309	18	0.72	0.30	0.97	–
Women	IL-2-pSTAT5-CD8 T cells	MCP.1	18	0.73	0.06	0.97	–
Women	IFNγ-pSTAT1-Monocytes	BDNF	18	0.74	-0.07	0.98	–
Women	IL-10-pSTAT3-Monocytes	Eotaxin	18	0.74	-0.17	0.98	–
Women	IFNγ-pSTAT1-Monocytes	RANTES	18	0.75	-0.02	0.98	–
Women	IL-2-pSTAT5-CD4 T cells	C5a	18	0.75	0.05	0.98	–
Women	IL-6-pSTAT1-CD4 T cells	C.TACK	18	0.75	-0.03	0.98	–
Women	IL-10-pSTAT3-B cells	sIL.6R	18	0.75	-0.07	0.98	–
Women	IFNα-pSTAT5-CD4 T cells	I.309	18	0.75	0.19	0.97	–
Women	IL-2-pSTAT5-CD8 T cells	C.TACK	18	0.76	-0.21	0.98	–
Women	IFNα-pSTAT5-CD4 T cells	I.TAC	18	0.76	-0.01	0.97	–
Women	IL-2-pSTAT5-CD4 T cells	RANTES	18	0.76	-0.07	0.97	–
Women	IFNα-pSTAT5-CD4 T cells	sIL.6R	18	0.76	-0.09	0.97	–
Women	IL-2-pSTAT5-CD4 T cells	IP.10	18	0.76	0.24	0.97	–
Women	IFNα-pSTAT3-CD8 T cells	C.TACK	18	0.77	-0.12	0.97	–
Women	IFNα-pSTAT5-CD4 T cells	Eotaxin	18	0.77	0.08	0.97	–
Women	IFNα-pSTAT1-CD4 T cells	IP.10	18	0.77	-0.13	0.97	–
Women	IFNα-pSTAT1-Monocytes	RANTES	18	0.78	-0.01	0.98	–
Women	IFNγ-pSTAT1-B cells	C5a	18	0.78	-0.01	0.98	–
Women	IL-6-pSTAT3-Monocytes	Eotaxin	18	0.78	0.26	0.98	–
Women	IL-6-pSTAT3-Monocytes	I.TAC	18	0.79	-0.03	0.97	–
Women	IL-10-pSTAT3-B cells	IL.6	17	0.79	-0.03	0.97	–

Table S8: *(continued)*

Sex	Stimulated condition	Marker	n	P value	rho	FDR	Selected
Women	IFNγ-pSTAT1-B cells	IL.10	17	0.79	-0.01	0.98	–
Women	IFNα-pSTAT1-B cells	I.TAC	18	0.79	0.12	0.98	–
Women	IL-6-pSTAT3-Monocytes	MIG	18	0.80	0.04	0.98	–
Women	IL-10-pSTAT3-CD8 T cells	P.sel	18	0.81	0.14	0.99	–
Women	IFNα-pSTAT1-B cells	P.sel	18	0.81	-0.02	0.99	–
Women	IFNα-pSTAT1-CD8 T cells	IL.6	17	0.82	-0.05	0.99	–
Women	IFNα-pSTAT3-B cells	BDNF	18	0.82	-0.23	0.99	–
Women	IFNα-pSTAT1-CD4 T cells	I.309	18	0.82	-0.07	0.99	–
Women	IFNα-pSTAT3-B cells	P.sel	18	0.82	0.02	0.99	–
Women	IL-10-pSTAT3-B cells	C5a	18	0.82	0.00	0.99	–
Women	IFNα-pSTAT1-Monocytes	BDNF	18	0.82	-0.22	0.98	–
Women	IFNα-pSTAT3-Monocytes	MIG	18	0.82	-0.09	0.98	–
Women	IFNα-pSTAT1-CD4 T cells	IL.10	17	0.83	0.05	0.98	–
Women	IL-10-pSTAT3-CD8 T cells	MCP.1	18	0.83	0.01	0.98	–
Women	IFNα-pSTAT3-B cells	C5a	18	0.84	0.07	0.99	–
Women	IL-10-pSTAT3-CD8 T cells	IP.10	18	0.85	0.06	0.99	–
Women	IL-10-pSTAT3-B cells	BDNF	18	0.85	-0.13	0.99	–
Women	IL-6-pSTAT3-Monocytes	GP130	17	0.85	-0.08	0.99	–
Women	IL-10-pSTAT3-CD4 T cells	MCP.1	18	0.85	-0.01	0.99	–
Women	IFNγ-pSTAT1-Monocytes	C5a	18	0.86	0.04	0.99	–
Women	IFNα-pSTAT1-CD8 T cells	sCD14	18	0.86	0.12	0.99	–
Women	IFNα-pSTAT1-CD4 T cells	CD40L	18	0.86	0.05	0.98	–
Women	IFNγ-pSTAT1-B cells	P.sel	18	0.86	-0.11	0.99	–
Women	IFNα-pSTAT3-Monocytes	sIL.6R	18	0.86	0.02	0.98	–
Women	IL-6-pSTAT3-Monocytes	IL.6	17	0.86	0.08	0.98	–
Women	IFNα-pSTAT3-B cells	IL.10	17	0.86	-0.11	0.98	–
Women	IFNγ-pSTAT1-Monocytes	IP.10	18	0.87	-0.06	0.98	–
Women	IFNγ-pSTAT1-B cells	sCD14	18	0.87	0.06	0.98	–
Women	IFNα-pSTAT5-CD4 T cells	MIG	18	0.87	-0.10	0.98	–
Women	IL-6-pSTAT1-CD4 T cells	sIL.6R	18	0.88	-0.13	0.99	–
Women	IL-10-pSTAT3-B cells	Eotaxin	18	0.89	-0.24	0.99	–
Women	IFNα-pSTAT3-Monocytes	C.TACK	18	0.89	0.10	0.99	–
Women	IL-10-pSTAT3-CD8 T cells	MIG	18	0.90	0.07	1.00	–
Women	IL-10-pSTAT3-B cells	IL.10	17	0.90	0.08	1.00	–
Women	IL-10-pSTAT3-Monocytes	MIG	18	0.90	0.02	1.00	–
Women	IL-10-pSTAT3-Monocytes	I.309	18	0.90	0.22	1.00	–
Women	IFNα-pSTAT1-CD8 T cells	IL.10	17	0.91	-0.09	1.00	–
Women	IFNα-pSTAT3-CD4 T cells	sIL.6R	18	0.92	-0.03	1.01	–
Women	IL-10-pSTAT3-CD4 T cells	MIG	18	0.92	0.01	1.01	–
Women	IL-10-pSTAT3-CD4 T cells	IL.10	17	0.93	0.07	1.01	–
Women	IL-6-pSTAT3-Monocytes	sCD14	18	0.93	0.00	1.01	–
Women	IFNα-pSTAT3-B cells	sIL.6R	18	0.93	-0.09	1.00	–
Women	IFNα-pSTAT3-CD4 T cells	IL.10	17	0.93	-0.05	1.00	–
Women	IFNα-pSTAT1-Monocytes	I.309	18	0.93	0.04	1.00	–
Women	IFNα-pSTAT3-CD4 T cells	GP130	17	0.93	-0.08	1.00	–
Women	IFNα-pSTAT3-Monocytes	CD40L	18	0.93	0.01	1.00	–
Women	IFNα-pSTAT3-B cells	IL.6	17	0.94	-0.06	1.00	–

Table S8: *(continued)*

Sex	Stimulated condition	Marker	n	P value	rho	FDR	Selected
Women	IFNγ-pSTAT1-B cells	I.309	18	0.94	0.04	1.00	–
Women	IFNα-pSTAT3-CD8 T cells	BDNF	18	0.95	-0.11	1.00	–
Women	IL-10-pSTAT3-CD4 T cells	IP.10	18	0.95	0.09	1.00	–
Women	IL-10-pSTAT3-B cells	I.309	18	0.95	0.09	1.00	–
Women	IFNα-pSTAT1-B cells	sIL.6R	18	0.96	-0.10	1.00	–
Women	IL-10-pSTAT3-CD8 T cells	I.309	18	0.96	0.17	1.01	–
Women	IFNα-pSTAT3-B cells	IP.10	18	0.96	-0.02	1.01	–
Women	IFNγ-pSTAT1-B cells	C.TACK	18	0.96	0.01	1.00	–
Women	IFNα-pSTAT5-CD4 T cells	P.sel	18	0.96	-0.08	1.00	–
Women	IFNγ-pSTAT1-Monocytes	P.sel	18	0.96	-0.03	1.00	–
Women	IFNγ-pSTAT1-B cells	CD40L	18	0.97	0.12	1.00	–
Women	IFNα-pSTAT3-CD8 T cells	sCD14	18	0.97	-0.02	0.99	–
Women	IL-10-pSTAT3-CD4 T cells	P.sel	18	0.98	0.08	1.01	–
Women	IFNα-pSTAT1-CD8 T cells	RANTES	18	0.98	0.01	1.00	–
Women	IFNα-pSTAT3-CD4 T cells	P.sel	18	0.98	-0.01	1.00	–
Women	IFNα-pSTAT3-CD4 T cells	CD40L	18	0.98	-0.16	1.00	–
Women	IFNα-pSTAT3-CD8 T cells	CD40L	18	0.99	-0.01	1.00	–
Women	IL-6-pSTAT3-Monocytes	CD40L	18	0.99	-0.06	1.00	–
Women	IL-10-pSTAT3-CD8 T cells	IL.10	17	0.99	0.10	1.00	–
Women	IL-6-pSTAT3-Monocytes	IP.10	18	1.00	0.06	1.00	–
Women	IFNα-pSTAT3-B cells	CD40L	18	1.00	-0.07	1.00	–
Women	IL-10-pSTAT3-CD4 T cells	GP130	17	1.00	0.07	1.00	–

Note:
Permutation version of the Spearman test was used. AUC: area under the concentration versus time curve per individual. 5 consecutive measurements per individual were performed over a timespan of 20 years. Tests are stratified by the 3 batches in which the phosflow measurements took place. FDR: (estimated) False Discovery Rate.

Table S9: Sex differences in cytokine levels after TLR stimulation

Stimulated condition	n	Direction	P value	FDR	Selected
LPS-TNFα	34	1	0.07	1.39	–
R848-TNFα	34	1	0.13	1.26	–
R848-IFNγ	34	-1	0.23	1.51	–
LPS-CXCL10	34	1	0.46	2.32	–
CpG-IL-6	34	-1	0.53	2.11	–
LPS-IL-10	34	-1	0.68	2.26	–
LPS-IL-8	33	1	0.68	1.95	–
R848-sGP130	34	-1	0.70	1.74	–
R848-IL-10	34	-1	0.72	1.60	–
R848-MCP1	34	-1	0.72	1.44	–
LPS-IL1β	34	1	0.75	1.36	–
LPS-sGP130	34	1	0.82	1.37	–
CpG-MCP1	34	-1	0.82	1.27	–
R848-CXCL10	34	1	0.82	1.18	–
R848-IL-8	34	-1	0.82	1.10	–
LPS-IFNγ	34	-1	0.85	1.06	–
LPS-IL-6	34	1	0.85	1.00	–
R848-IL-6	34	1	0.85	0.95	–
R848-IL1β	34	1	0.91	0.95	–
LPS-MCP1	34	1	0.96	0.96	–

Note:
Cytokine levels are expressed as fold change compared to unstimulated levels. Permutation version of the Wilcoxon-Mann-Whitney test was used. FDR: (estimated) False Discovery Rate.

Table S10: Associations of cytokine levels after TLR stimulation with frailty in men and women

Sex	Stimulated condition	n	P value	rho	FDR	Selected
Men	CpG-MCP1	16	0.03	-0.55	0.57	–
Men	R848-IFNγ	16	0.03	-0.55	0.31	–
Men	R848-CXCL10	16	0.03	-0.54	0.21	–
Men	LPS-CXCL10	16	0.12	-0.40	0.62	–
Men	R848-IL1β	16	0.16	-0.37	0.63	–
Men	R848-sGP130	16	0.17	-0.36	0.56	–
Men	R848-MCP1	16	0.19	-0.34	0.56	–
Men	LPS-sGP130	16	0.23	-0.32	0.57	–
Men	R848-IL-8	16	0.26	-0.30	0.57	–
Men	CpG-IL-6	16	0.26	-0.30	0.53	–
Men	LPS-MCP1	16	0.29	-0.28	0.53	–
Men	LPS-IL-8	15	0.33	-0.27	0.55	–
Men	LPS-IFNγ	16	0.34	-0.25	0.53	–
Men	LPS-IL-6	16	0.34	-0.25	0.49	–
Men	R848-IL-6	16	0.36	-0.24	0.48	–
Men	R848-IL-10	16	0.37	-0.24	0.46	–
Men	LPS-IL1β	16	0.56	-0.16	0.66	–
Men	R848-TNFα	16	0.64	-0.12	0.71	–
Men	LPS-IL-10	16	0.89	-0.04	0.93	–
Men	LPS-TNFα	16	0.99	0.00	0.99	–
Women	R848-IL-8	18	0.15	0.35	2.99	–
Women	R848-IFNγ	18	0.18	-0.33	1.85	–
Women	R848-MCP1	18	0.31	0.25	2.09	–
Women	LPS-IL-10	18	0.32	-0.25	1.61	–
Women	R848-CXCL10	18	0.33	-0.24	1.34	–
Women	R848-sGP130	18	0.37	0.22	1.24	–
Women	R848-IL1β	18	0.45	-0.19	1.29	–
Women	CpG-MCP1	18	0.48	-0.18	1.20	–
Women	LPS-CXCL10	18	0.50	-0.17	1.11	–
Women	CpG-IL-6	18	0.55	-0.15	1.09	–
Women	LPS-IFNγ	18	0.63	-0.12	1.14	–
Women	LPS-sGP130	18	0.68	0.10	1.13	–
Women	LPS-MCP1	18	0.70	0.10	1.08	–
Women	LPS-IL-8	18	0.70	0.10	1.00	–
Women	R848-IL-10	18	0.73	-0.09	0.97	–
Women	R848-IL-6	18	0.77	0.07	0.97	–
Women	LPS-IL-6	18	0.80	0.06	0.94	–
Women	LPS-IL1β	18	0.85	-0.05	0.94	–
Women	R848-TNFα	18	0.94	-0.02	0.99	–
Women	LPS-TNFα	18	0.96	-0.01	0.96	–

Note:
Cytokine levels are expressed as fold change compared to unstimulated levels. Permutation version of the Wilcoxon-Mann-Whitney test was used. FDR: (estimated) False Discovery Rate.
